# Supplementary material for: Efficacy of acupuncture in refractory irritable bowel syndrome: study protocol for a randomised controlled trial
Source: BMJ Open. 2021 Sep 13;11(9):e045655. doi: 10.1136/bmjopen-2020-045655 (PMC8438864; doi:10.1136/bmjopen-2020-045655)
Supplement: Author's manuscript [file bmjopen-2020-045655.draft_revisions.pdf]

BMJ Open

BMJ Open is committed to open peer review. As part of this commitment we make the peer review history of every article we publish publicly available.

When an article is published we post the peer reviewers' comments and the authors' responses online. We also post the versions of the paper that were used during peer review. These are the versions that the peer review comments apply to.

The versions of the paper that follow are the versions that were submitted during the peer review process. They are not the versions of record or the final published versions. They should not be cited or distributed as the published version of this manuscript.

BMJ Open is an open access journal and the full, final, typeset and author-corrected version of record of the manuscript is available on our site with no access controls, subscription charges or pay-per-view fees (http://bmjopen.bmj.com).

If you have any questions on BMJ Open's open peer review process please email info.bmjopen@bmj.com

BMJ Open

Efficacy of acupuncture in refractory irritable bowel syndrome: study protocol for a randomised controlled trial

Journal:	*BMJ Open*
Manuscript ID	bmjopen-2020-045655
Article Type:	Protocol
Date Submitted by the Author:	12-Oct-2020

Complete List of Authors:	Zhao, Jun; Beijing University of Chinese Medicine, School of Acupuncture-Moxibustion and Tuina<br>Chen, Min; Chengdu University of Traditional Chinese Medicine Affiliated Hospital<br>Wang, Xin; Capital Medical University Affiliated Beijing Hospital of Traditional Chinese Medicine<br>Ye, Kun; Chengdu University of Traditional Chinese Medicine, Chengdu, Sichuan, China<br>Shi, Suhua; Department of Rehabilitation, The Third Affiliated Hospital of Beijing University of Chinese Medicine, Beijing, China<br>Li, Huixia; Department of Gastroenterology, Third Affiliated Hospital of Beijing University of Chinese Medicine, Beijing, China<br>Wang, Jianfang; Department of Spleen, Stomach, Liver and Gallbladder Diseases, Dongfang Hospital Affiliated to Beijing University of Chinese Medicine, Beijing, China<br>Chen, Xiaowei; Department of Spleen, Stomach, Liver and Gallbladder Diseases, Dongfang Hospital Affiliated to Beijing University of Chinese Medicine, Beijing, China<br>Ni, Jinxia; Department of Acupuncture and Moxibustion, Dongzhimen Hospital Affiliated to Beijing University of Chinese Medicine, Beijing, China<br>Wei, Qingshuang; School of Acupuncture-Moxibustion and Tuina, Beijing University of Chinese Medicine, Beijing, China<br>Shi, Yunzhou; Acupuncture and Tuina School, Chengdu University of Traditional Chinese Medicine<br>Hu, Yu; School of Acupuncture-Moxibustion and Tuina, Beijing University of Chinese Medicine, Beijing, China<br>Sun, Jingwen; School of Acupuncture-Moxibustion and Tuina, Beijing University of Chinese Medicine, Beijing, China<br>Li, Da; Department of Acupuncture and Moxibustion, Dongzhimen Hospital Affiliated to Beijing University of Chinese Medicine, Beijing, China<br>Liu, Siyuan; School of Acupuncture-Moxibustion and Tuina, Beijing University of Chinese Medicine, Beijing, China<br>Li, Zhigang; Beijing University of Chinese Medicine, School of Acupuncture-moxibustion and Tuina<br>Zheng, Hui ; Chengdu University of Traditional Chinese Medicine,<br>Yu, Shu-guang; Chengdu University of Traditional Chinese Medicine, School of Acupuncture and Tuina

Keywords:	COMPLEMENTARY MEDICINE, Gastroenterology < INTERNAL MEDICINE,

	Protocols & guidelines < HEALTH SERVICES ADMINISTRATION & MANAGEMENT

I, the Submitting Author has the right to grant and does grant on behalf of all authors of the Work (as defined in the below author licence), an exclusive licence and/or a non-exclusive licence for contributions from authors who are: i) UK Crown employees; ii) where BMJ has agreed a CC-BY licence shall apply, and/or iii) in accordance with the terms applicable for US Federal Government officers or employees acting as part of their official duties; on a worldwide, perpetual, irrevocable, royalty-free basis to BMJ Publishing Group Ltd ("BMJ") its licensees and where the relevant Journal is co-owned by BMJ to the co-owners of the Journal, to publish the Work in this journal and any other BMJ products and to exploit all rights, as set out in our licence.

The Submitting Author accepts and understands that any supply made under these terms is made by BMJ to the Submitting Author unless you are acting as an employee on behalf of your employer or a postgraduate student of an affiliated institution which is paying any applicable article publishing charge ("APC") for Open Access articles. Where the Submitting Author wishes to make the Work available on an Open Access basis (and intends to pay the relevant APC), the terms of reuse of such Open Access shall be governed by a Creative Commons licence – details of these licences and which Creative Commons licence will apply to this Work are set out in our licence referred to above.

Other than as permitted in any relevant BMJ Author's Self Archiving Policies, I confirm this Work has not been accepted for publication elsewhere, is not being considered for publication elsewhere and does not duplicate material already published. I confirm all authors consent to publication of this Work and authorise the granting of this licence.

For peer review only

Efficacy of acupuncture in refractory irritable bowel syndrome: study protocol for a randomised controlled trial

Jun Zhao,[1] Min Chen,[2] Xin Wang,[3] Kun Ye,[4] Suhua Shi,[5] Huixia Li,[6] Jianfang Wang,[7] Xiaowei Chen,[7] Jinxia Ni,[8] Qingshuang Wei,[1] Yunzhou Shi,[4] Yu Hu,[1] Jingwen Sun,[1] Da Li,[1,8] Siyuan Liu,[1] Zhigang Li,[1] Hui Zheng,[4] Shu-Guang Yu[4]

JZ, MC, and XW contributed equally.

Correspondence to

Zhigang Li, School of Acupuncture-Moxibustion and Tuina, Beijing University of Chinese Medicine, Beijing, China (e-mail:lizhigang620@126.com).

Hui Zheng, Chengdu University of Traditional Chinese Medicine, Chengdu, Sichuan, China (e-mail: zhenghui@cdutcm.edu.cn).

Shu-Guang Yu, Chengdu University of Traditional Chinese Medicine, Chengdu, Sichuan, China (e-mail: ysg@cdutcm.edu.cn).

ABSTRACT

Introduction Irritable bowel syndrome (IBS) is a common chronic functional gastrointestinal disorder that presents with abdominal pain /discomfort and altered bowel patterns. Due to IBS has multiple potential causes for which conventional medicines have had limited success, resulting in a significant amount of patients who do not sensitively respond to pharmacotherapy for a period of 12 months and develop a continuing symptom profile (described as refractory IBS) seeking help through (non) pharmacological treatments. The aim of this study is to investigate the efficacy and safety of acupuncture therapy for refractory IBS on the basis of conventional treatments.

Methods and analysis A total of 170 eligible patients who meet the Rome IV criteria for refractory IBS will be randomly allocated to receive acupuncture or sham acupuncture. Each patient will receive 12 sessions of acupuncture over 4 weeks and a 4-week follow-up. The primary outcome is the IBS-symptom severity score (IBS-SSS). Secondary outcomes include the proportion of participants experiencing adequate relief of global IBS symptoms, the frequency of defecation weekly, the stool property bristol grading score, the work and social adjustment score (WSAS), the IBS-quality of life (IBS-QOL) score, the self-rating depression scale (SDS) and self-rating anxiety scale (SAS) anxiety and depression scores. Outcome measures are collected at baseline, 2 weeks and 4 weeks of the intervention, 6 weeks and 8 weeks after the intervention. Categorical variables will be compared with the Fisher's exact test or Wilcoxon rank-sum test, and continuous variables will be compared using the Student's t-test or Wilcoxon rank-sum test.

Ethics and dissemination The entire project has been approved by the ethics committee of Beijing University of Chinese Medicine (2020BZYLL0507) and sichuan province regional institutional for conducting research of Traditional Chinese Medicine (2020KL-025). The outcomes of the trial will be disseminated through peer-reviewed publications.

Trial registration number NCT04276961.

For peer review only - http://bmjopen.bmj.com/site/about/guidelines.xhtml

Strengths and limitations of this study

- This is the first multicentre randomized controlled trial verifying the added benefit of acupuncture to usual treatments for refractory IBS.
- In view of the complex psychological feelings of refractory patients, the acupuncture treatment evaluation will be conducted in clinical settings where usual treatments are routinely carried out.
- This trial meets the methodological demand of adequate concealment of randomised group allocations, outcome assessors, and statisticians.
- A limitation of this trial is that since a rigorous study design was developed to identify which patients with IBS are "refractory", we must fully consider the differences in the severity of symptoms, the duration of symptoms, the dietary interventions or advice provided, and even the conventional pharmaceutical treatments provided.

INTRODUCTION

Irritable bowel syndrome (IBS) is the most commonly functional gastrointestinal disorder worldwide.[1] Epidemiological data shows that the prevalence of IBS is approximately 5-6% in China,[2] 9.5-9.8% in Asia,[3] and 11% in the world,[4] which indicates that IBS is a major public health issue. Owing to the complex pathogenesis of IBS, several pathogenic factors (genetic and environmental factors, visceral hypersensitivity, abnormal gut motility, post-infectious inflammatory mechanisms, gut microbiota changes, psychological morbidity, and physical and emotional factors) have been proposed as possible causes.[5] Hence, the study of patients with IBS is still a challenge.

Furthermore, in clinical practice, IBS patients complain about various symptoms, such as changes in bowel habits, abdominal pain, diarrhoea or constipation, and meteorism, which considerably worsen their quality of life. The Rome IV criteria suggest that IBS treatment is selected on the basis of the symptom type (abdominal pain, diarrhoea, etc.).[1] Simultaneously, patients are stratified into four subtypes: constipation-predominant IBS (IBS-C), diarrhea-predominant IBS (IBS-D), mixed IBS (IBS-M), and unclassified IBS (IBS-U). Many medications for treating IBS-related symptoms have been developed for various IBS subtypes, including antispasmodics,

For peer review only - http://bmjopen.bmj.com/site/about/guidelines.xhtml

psychotropic agents, bulking agents, 5-HT receptor antagonists, antibiotics, probiotics and so on. Although 5-HT3 antagonists, 5-HT4 agonists, antispasmodics, and alosetron have been shown to be useful for the treatment of IBS,[6] regretfully, some drugs, such as guanylate cyclase-C agonists and loperamide, have been proven to be inadequate for relieving symptoms.[6 7] Similarly, some psychological therapies, such as hypnotherapy, relaxation therapy and cognitive-behavioural therapy, have been proven to be effective treatments for IBS.[8] Even when these above available treatments are used, symptoms can persist for more than 12 months in some patients with IBS. These patients with IBS can be considered as therapy resistant (refractory).[9 10] Therefore, novel treatment options for IBS, especially refractory IBS, are urgently needed.

Recently, there has been an increase in the use of non-pharmacological therapies, including acupuncture, which is widely accepted as an effective alternative treatment for gastrointestinal disorders in clinical practice.[11] According to reviews of the literature, acupuncture was not only satisfactory for IBS, but also may be more effective than pharmacological therapies in alleviating the symptoms and quality of life, with its effects lasting up to 12 weeks,[12] even sustaining benefits over a period of 12 months post-randomisation.[13 14] However, there are still limitations with previous researchs, including small sample sizes, poor quality of the trials and lack of detailed assessment of symptom improvement, such as social activities, psychological questionnaire, etc. Meanwhile, rare studies have been designed for refractory IBS with acupuncture. The main objective of this trial is to evaluate, on the basis of conventional treatments, the efficacy of acupuncture versus sham acupuncture for refractory IBS in terms of symptom management.

METHODS/DESIGN

Study design

This multicenter randomized controlled trial will be conducted at 6 hospitals in China: Dongzhimen Hospital Affiliated to Beijing University of Chinese Medicine; Dongfang Hospital Affiliated to Beijing University of Chinese Medicine; Third Hospital

For peer review only - http://bmjopen.bmj.com/site/about/guidelines.xhtml

Affiliated to Beijing University of Chinese Medicine; Beijing Hospital of Traditional Chinese Medicine Affiliated to Capital Medical University; Clinical Medicine College/Teaching Hospital Affiliated to Chengdu University of Traditional Chinese Medicine, and Third Hospital/Acupuncture and Tuina School Affiliated to Chengdu University of Chinese Medicine. The study has been approved by ethics committees and registered on at the www.clinicaltrials.gov on 19 February 2020 (Registration number: NCT04276961). The study protocol will follow the principles of the Declaration of Helsinki and clinical research to Good Clinical Practice (GCP) guidelines, and will be reported based on SPIRIT 2013 Checklist.[15] The checklist is provided in the online supplementary additional file 1.

The eligible patients will be randomly divided into two groups, among participants who will receive 1 week of wash-out, 12 sessions over 4 weeks of treatment, and 4 weeks of post-treatment follow-up. Outcomes will be assessed at baseline, during the treatment and at the end of the follow-up. A flow diagram of the trial is shown in figure 1.

Patient recruitment

Step 1, patients meeting the Rome IV IBS criteria (table 1) will be primarily recruited from the outpatient centers of the 6 participating hospitals. The mode of recruitments also including through advertisements on hospital social Internet media (WeChat subscription), posters, sojump and flyers about this trial, outpatient clinics, and publicity at surrounding community service centers. Step 2, patients with 1) IBS-related symptoms lasting for at least 12 months 2) and without a response to currently available IBS treatments, such as antibiotics or probiotics, dietary changes, antidepressants, or psychotherapies will be included.[16-18] Clinicians will primarily select the patients with suspected refractory IBS for further detailed assessment. Step 3, a clinical research assistant will identify eligible participants for this trial according to the following rigorous inclusion and exclusion criteria (table 2 and table 3). All patients will voluntarily agree to participate and sign informed consent document before

For peer review only - http://bmjopen.bmj.com/site/about/guidelines.xhtml

randomization.

Table 1 Rome IV criteria: irritable bowel syndrome [19]

Diagnostic time frame	Symptom description	Symptoms
① ≥ 3 months of persistent symptoms with symptom onset at least 6 months before diagnosis<br>② ≥ 1 day per week	Abdominal pain	Recurrent association (2 or more) of the following criteria:<br>①Related to defecation<br>② Associated with a change in the form of stool<br>③ Associated with a change in the frequency of stool

Table 2 Inclusion criteria

Items	
①	Adults aged 18-80 years (either sex);
②	Fulfilled Rome IV criteria for IBS;
③	Symptoms are present for ≥12 months;
④	There is an absence of response to an adequate dose of at least one conventional pharmacological agent tried for a minimum of 6 weeks;
⑤	There is an absence of response to a minimum of 6 weeks of dietary intervention or advice;
⑥	Abdominal pain score ≥40 mm for at least 1 day in a week;
⑦	The results of colonoscopy are normal in near year;
⑧	Signed the written informed consent form.

For peer review only - http://bmjopen.bmj.com/site/about/guidelines.xhtml

Table 3 Exclusion criteria

Items	
①	Diagnosis of an organic intestinal disease (including but not limited to ulcerative colitis, familial multiple intestinal polyps, colorectal carcinoma);
②	The presence of one or more of the following warning symptoms: unexplained rectal bleeding, a positive faecal occult blood test result; anaemia, abdominal mass, ascites, fever, and emaciation;
③	The presence of other severe medical conditions, such as cardiovascular disease, endocrine disorders, hepatic dysfunction, renal diseases, and cognitive disorders that can affect the outcome assessment results;
④	An unstable psychological state or accompanying psychological disorders (SDS>56);
⑤	With pregnancy or lactation;
⑥	Accepting acupuncture treatment in the last 3 months;
⑦	Difficulties in attending the trial, such as fear of acupuncture.

Randomization/allocation

A blocked randomization sequence for multicenter will be generated by an independent

For peer review only - http://bmjopen.bmj.com/site/about/guidelines.xhtml

For peer review only

professional statistician (Linkermed Tech Co., Ltd), using Statistical Analysis System (SAS) software. And all eligible participants will be randomly stratified into the acupuncture group or sham acupuncture group in a 1:1 ratio. Simultaneously, we plan to cooperate with a remote central web-based randomization system. Whereas the clinical research assistant will be responsible for enrolling patients, screening patients, obtaining informed consent, requesting randomization and data collection.

Blinding

Patients as well as research assistant, outcome assessors and statisticians will be blinded to group assignment. However, due to the nature of acupuncture, the acupuncturists providing the interventions cannot be blinded. To minimize the known bias sources, the number/or location of the acupuncture points and manipulation of needles are similar in the acupuncture or sham acupuncture group, thereby optimizing the blinding of subjects. Similarly, to avoid the subjective bias of researchers and subjects, the data analysis of the whole experiment will adopt the third-party blind evaluation. And the participant's allocated intervention will be not revealed until the statistical analysis reportis finalized.

Interventions

In order to ensure the safety and proper operation of acupuncture. All the acupuncturists will participate in the standard operation process (SOP) of the skin disinfection, location acupoints and non-acupoints, fundamental puncture and needles manipulations before the trials. And treatments will be performed by licensed acupuncturists who have at least 3 years of experience in acupuncture. Acupuncture will be discontinued if the patients suffer from any cases of adverse event (AE) after acupuncture. The schedule of enrolment, interventions, assessments, and participant visits is shown in figure 2.

Treatment as usual (TAU)

TAU is defined as the continuation of current therapeutic agents prescribed by a general

For peer review only - http://bmjopen.bmj.com/site/about/guidelines.xhtml

practitioner or gastroenterologist. All patients will continue TAU during the study period. Any changes in medications of individual participant will be recorded by diary card.

Acupuncture group

Patients allocated to the acupuncture group, with disposable and sterile acupuncture needles (length: 25-40 mm, diameter: 0.30 mm; Hwatuo, Suzhou, China), will take treatment acupuncture three times a week for 4 weeks for a total of 12 treatment sessions. Based on Traditional Chinese Medicine (TCM) theory and our clinical experience, the prespecified acupoints used here included bilateral *Tianshu (ST25), Shangjuxu (ST37), Zusanli (ST36), and Neiguan (PC6)* (table 4 and figure 3). The exact location and depth of needling for each point will be determined based on the 2006 People's Republic of China National Standard: The Name and Location of Acupoints (GB/T 12346-2006).[20] After insertion, manipulations of twirling, lifting, and uniform reinforcing-reducing manipulation will be performed on all needles to elicit "deqi". The compositional sensation of "deqi" includes numbness, soreness, distention, aching, and heaviness, which are believed to be an essential component for acupuncture efficacy.[21] Then removed the needles after 30-min session with clean cotton balls to avoid bleeding.

Table 4 Locations of acupoints in acupuncture group

Acupoints	Locations
Tianshu (ST25)	On the same level of the umbilicus, and 2 cun* lateral to the anterior midline
Shangjuxu (ST37)	6 cun directly below Dubi (ST35), and one finger-breadth lateral to the anterior border of the tibia
Zusanli (ST36)	3 cun directly below Dubi (ST35), and one finger-breadth lateral to the anterior border of the tibia
Neiguan (PC6)	On the line joining Daling (PC7) and Quze (PC3), between the tendons of palmaris longus and flexor carpi radialis, 2 cun above the transverse crease of the wrist

For peer review only - http://bmjopen.bmj.com/site/about/guidelines.xhtml

*1 cun (≈20 mm) is defined as the width of the interphalangeal joint of patient's thumb

Sham acupuncture group

The participants in the sham acupuncture group will also receive the same treatment duration and frequency of sessions as the real acupuncture group. Similarly, sham acupuncture at predefined sites not corresponding to acupuncture group traditional points or meridians, penetrating to the same depth but without needle manipulation for "deqi" (table 5 and figure 3). Patients will be informed of the 2 types of acupuncture as follows: "in this trail, different types of acupuncture will be compared but another approach might not target your additional symptoms." This language will be used to avoid deception while achieve unify the simulation conditions of two groups as far as possible, then mix real and sham points, and might enhance patient compliance.

Table 5 Locations of non-acupoints in sham acupuncture group

Non-acupoints	Locations
Non-acupoint 1	In the middle of Daheng (SP15) and Zhangmen (LR13) points (lateral aspect)
Non-acupoint 2	In the medial arm on the anterior border of the insertion of the deltoid muscle at the junction of the deltoid and biceps muscles
Non-acupoint 3	On the same level 1-2 cun outboard lateral of the ST36
Non-acupoint 4	In the middle of the medial epicondyle of the humerus and the styloid process of ulna

*1 cun (≈20 mm) is defined as the width of the interphalangeal joint of patient's thumb

Outcomes

Primary outcome

IBS symptom severity score (IBS-SSS)

For peer review only - http://bmjopen.bmj.com/site/about/guidelines.xhtml

According to previous assessment recommendations,[22] IBS symptom severity score (IBS-SSS) is a validated scoring system, which relates to the severity of abdominal pain (IBS-SSS1), frequency of abdominal pain (IBS-SSS2), bloating (IBS-SSS3), satisfaction with bowel habits (IBS-SSS4), and disruption quality of life (IBS-SSS5), will be used as the primary outcome of this trial. Participants will respond to each question on a 100-point visual analogue scale (VAS). Each of the 5 questions generated a maximum score of 100 points, and the total is scored from 0 to 500 with higher scores indicating severe symptoms: normal (< 75), mild IBS case (75-175), moderate IBS case (175-300), and severe IBS case (>300). IBS-SSS will be assessed at baseline, 2, 4, 6 and 8 weeks after randomization.

Secondary outcomes

Adequate relief of global IBS symptoms

During our treatment period, patients will respond weekly to the question: "In regard to all your symptoms of IBS, as compared with the way you felt before you added acupuncture therapy on the basis of conventional treatments, have you, in the past 7 days, had adequate relief of your IBS symptoms?" (yes/no). "Yes" will be regarded as a positive response to treatment.[23]

Bristol Stool Form Scale

The Bristol stool form scale classifies stool consistency into 7 types encompassing complications from constipation to diarrhea.[24] In this trial, participants will be asked to record the type and score of defecations in a week. The scores obtained from Bristol scale will be assessed at baseline, weeks 2, 4, 6 and 8.

The frequency of defecation weekly

The frequency of bowel movements during a week before each visit will be assessed at baseline, 2, 4, 6 and 8 weeks after randomization. A normal defecation is defined as a daily stool frequency ≤ 3 times and stool consistency to be Type 4 (Like a sausage or snake, smooth and soft).[25]

For peer review only - http://bmjopen.bmj.com/site/about/guidelines.xhtml

Work and Social Adjustment Scale (WSAS)

The WSAS will be assessed at baseline, weeks 2, 4, 6 and 8 using 5-domain scale with a total possible score of 40, of each item scores 0 (not affected) to 8 (severely affected), respectively. It is used to measure problems in functioning with work, home management, social activities, private leisure activities, and family and relationships.[26]

IBS-Quality of Life (IBS-QOL)

The IBS-QOL questionnaire consists of 34 items, each item includes 5-point response scale, is a valid and reliable instrument to establish changes in health-related QOL patients with IBS.[27] Patients themselves will be required to fill the questionnaire at baseline, weeks 2 and 4 after the treatment, respectively. Then total scores of each item will be calculated and analyzed with higher scores suggestive of better QOL.

Self-Rating Anxiety Scale (SAS) and Self-Rating Depression Scale (SDS)

The majority of IBS patients have comorbid anxiety and/or depression, which are in turn strongly associated with symptoms severity.[28 29] Hence, anxiety and depression symptoms will be assessed at baseline, weeks 2 and 4 using the SAS and SDS. It is noteworthy that (SDS>56) will be excluded before the first treatment because of an unstable psychological state or accompanying psychological disorders. According to statistical standard, the total score is multiplied by 1.25 and is then converted into a standardized score ranging from 25 to 100, For SAS, the grade 1, 2 and 3 are related with 53-62, 63-72 and >72 scores, respectively.[30] For SDS, grade 1, 2 and 3 are for 50-60, 61-70 and >70 scores, respectively.[31] Higher scores indicate more severe anxiety and depression.

Blinding assessment

To test the patient-blinding effects, all patients will be asked to guess whether they have received acupuncture or sham acupuncture at the end of the first treatment session and the twelfth treatment session.

For peer review only - http://bmjopen.bmj.com/site/about/guidelines.xhtml

Safety evaluation

In this trail, AEs will be monitored during the 4 weeks of treatment and next 4 weeks follow-up. Any adverse reactions occurred during the study will be recorded. Common side effects of acupuncture or sham acupuncture will include subcutaneous hematoma, local bleeding, skin bruising, needle site itching, continuous needle site pain, muscle spasm, dizziness, and so on. In the event of a serious adverse event (SAE), the necessary measures will be taken immediately for the safety of the subject. The time of occurrence, severity, duration, outcome, and treatment-related or not will be reported to the Medical Ethics Committee within 24 h.

Sample size and statistical analysis

Based on two samples size mean literature data speculation and previous clinical experience,[12 32] we anticipate the scores of two acupuncture group on the continuation of current medications, responders can improve refractory IBS on the IBS-SSS will be 30 and 15, respectively. The standard deviation is set at 15, and the ratio between the acupuncture and sham acupuncture groups is 1:1. The sample size is calculated with 20% type II error rate (80% power) and 5% type I error. The superiority trial will be adopted to minimize of the two groups difference, and a 10-point is expected. In total, 154 (77 patients in each group) is estimated. Allowing for 10% loss to follow-up, the sample size expands to 85 patients in each group.

All analyses will be performed using the intent-to-treat (ITT) and the per-protocol (PP) population. ITT requires all participants who receives baseline assessment of primary outcome and at least 1 acupuncture session or 1 sham acupuncture administration. Missing values will be imputed through the multiple imputation method. Whereas the PP population is usually defined as patients who completing at least 80% of the treatment protocol without major protocol violations. The primary outcome will be assessed using analysis of covariance (ANCOVA) and adjusted for baseline total IBS-SSS. For other secondary outcomes: continuous variables will be analysed using the Student's t-test or Wilcoxon rank-sum test, and data will be represented as the mean ± SD or median; Categorical variables will be calculated using Fisher's exact test or

For peer review only - http://bmjopen.bmj.com/site/about/guidelines.xhtml

Wilcoxon rank-sum test, and data will be represented by frequency (percentage). In terms of efficacy factors, other covariates such as age, gender, disease duration, conventional pharmacological agent classification and so on will be considered for further revision. The differences in blinding and adverse events between the groups will be compared using the chi-square test. Meanwhile, we decide to carry out subgroup analysis based on patients with IBS subtypes, avoiding a heterogeneous patient population which may affect the efficacy results.

Statistical analysis of all data in this study will be performed by a specialized third-party statistician evaluation. SAS, version 8.2 statistical software (SAS Institute Inc) will be used for data analysis. A two-sided $P<0.05$ is considered statistically significant.

Data management and quality control

To guarantee the objectivity of the data and individual participant data will not be disclosed outside. All researchers will receive training regarding data management. Data entry and management will be conducted using a database software clinical trial data management system by data administrators of China academy of Chinese medical science. An electronic case report form (eCRF) will be designed to collect all clinical observation results of each participant before recruitment. The research assistant is responsible for verifying the accuracy and integrity of data to prevent any detections of errors, omissions, or items requiring changes or clarification. Data lockup will be implemented by the data administrators on completion of the study. All paper files and electronic data will be kept for 5 years after publication and destroyed after then.

To guarantee the quality of this trial, prespecified SOP including recruitment and screening of participants, randomization, laboratory detection results, intervention, details in filling CRF, assessment of outcomes, data management, and AEs of acupuncture will be unified trained through multicenters internal study for consistency of results, respectively. Moreover, we will set up a Data and Safety Monitoring Board, an independent advisory group, to review and interpret data generated from the study, and a meeting will be held every 3 months to report the research progress. Each amendment of the protocol conforms to the GCP principles and maintains ethical

For peer review only - http://bmjopen.bmj.com/site/about/guidelines.xhtml

standards of the RCT.

Patients and public involvement

Patients in this trial will not involved in the design or conduct of the study or the outcome assessments. However, we are planning to disseminate our research to the participants and the public, such as publicising our research in hospital social Internet media (WeChat subscription) and various academic lectures.

Ethics and dissemination

This study conforms to the principles of the Declaration of Helsinki and relevant ethical guidelines. The entire project has been approved by the ethics committee of Beijing University of Chinese Medicine (ID: 2020BZYLL0507) and sichuan province regional institutional for conducting research of Traditional Chinese Medicine (ID: 2020KL-025). Written informed consent will be obtained from patients prior to enrolment in the study. The outcomes of the trial will be disseminated through peer-reviewed publications.

Discussion

IBS not only impacts patients' health-related QOL, resulting in a large economic burden on healthcare systems, but also impairs work productivity and individuals' ability to perform daily activities, increasing employers' indirect costs.[33] The currently available pharmacological treatments still have some limitations, resulting in many refractory IBS patients seeking help through complementary and alternative medicine.[34]

However, when considering refractory IBS treatment, the symptoms should be highly addressed to maintain optimal results and enhance the quality of life of individuals. The IBS, especially refractory IBS had more severe psychosomatic disorders.[35] Patients who classified with severe IBS agreed with the statement: 'When my IBS is bad, I wish I was dead'.[36] Hence, another important treatment principle for refractory IBS is improving mental/emotional problems. From a TCM perspective,

For peer review only - http://bmjopen.bmj.com/site/about/guidelines.xhtml

acupoint selection is a key factor in the success of acupuncture in treating diseases. According to previous studies and our previous reviews, the basic acupuncture points important for relieving diarrhoea symptoms are ST25, ST36 and ST37.[37 38] However, acupuncture treatment for psychosomatic diseases highlights the specific functions of acupoints. Clinical evidence has shown that PC6 can improve anxiety symptoms well.[39] Hence, the ST25, ST36, ST37 and PC6 points included in this trial are selected in accordance with not only the TCM treatment principles of "syndrome differentiation" but also clinical practice guidelines in China. However, for these points, the scientific evidence is sparse, and high-quality research is often lacking, leading to inconclusive results.[34] As such, a multicentre RCT with a large sample size is urgently needed to evaluate the efficacy of acupuncture in improving refractory IBS symptoms within the evidence-based medical framework.

In view of the complex psychological feelings of refractory patients, the acupuncture treatment evaluation will be conducted in clinical settings in which usual treatment is routinely carried out. The many benefits of TAU include surmounting critical ethical issues (e.g., of withholding treatment),[40] whereas controlling for many common factors (e.g., psychological concerns, dietary factors, and medication changes) that can contribute to change. What's more, owing to there is uncertain whether the long-term clinical benefit of acupuncture on refractory IBS is sustained, another possible advantage of TAU is that research staff could establish good communication relationships with patients, thus avoiding potential dropouts. In this way, can we cautiously extrapolate the long-term effect of acupuncture.

Nevertheless, this study still has several limitations that should be noted. First, identifying which patients with IBS are "refractory" is challenging because of the differences in the severity of symptoms, usual treatments administered and duration of symptoms.[41] On the basis of a literature review, refractory IBS patients were defined as those with an average daily pain rate ≥30 mm on the pain component scale of the IBS-SSS,[22] who failed to respond to usual medical treatment (education, dietary advice, antispasmodic agents, antidiarrhoeal medication, antidepressants, or fibre-based medications, etc.),[16-18 42 43] and who had IBS symptoms for 12 months or more.[9 10 43-45]

For peer review only - http://bmjopen.bmj.com/site/about/guidelines.xhtml

Second, to ensure a reliable sample of refractory IBS patients, we plan to conduct two rounds of screening: in the first step, the clinicians will primarily select patients with suspected refractory IBS according to the above literature review items; then, a clinical research assistant will recommend the eligible participants for this trial according to the improved critical inclusion criteria 1) there is an absence of response to an adequate dose of at least one conventional pharmacological agent tried for a minimum of 6 weeks; 2) there is an absence of response to a minimum of 6 weeks of dietary intervention or advice and 3) abdominal pain score ≥40 mm for at least 1 day in a week. Moreover, we attach great importance to the potential risk of symptoms in refractory patients. The following warning symptoms (one or more): unexplained rectal bleeding, a positive faecal occult blood test result; anaemia, abdominal mass, ascites, fever, and emaciation will be excluded. All participants will have to fill in the "Filter table" in advance to guarantee that all inclusion criteria will be met.

At the end of this trial, we hope the results will provide more reliable evidence and reveal the value of clinical acupoint selection guidelines in the treatment of refractory IBS with acupuncture.

TRIAL STATUS

This trial is currently in the preparation phase. No patient involved.

Author affiliations

[1]School of Acupuncture-Moxibustion and Tuina, Beijing University of Chinese Medicine, Beijing, China
[2]Colorectal Department/ Clinical Medicine College, Hospital of Chengdu University of Traditional Chinese Medicine, Chengdu, China
[3]Department of Acupuncture and Moxibustion, Beijing Hospital of Traditional Chinese Medicine, Capital Medical University, Beijing, China
[4]Chengdu University of Traditional Chinese Medicine, Chengdu, Sichuan, China
[5]Department of Rehabilitation, Third Affiliated Hospital of Beijing University of Chinese Medicine, Beijing, China
[6]Department of Gastroenterology, Third Affiliated Hospital of Beijing University of Chinese Medicine, Beijing, China
[7]Department of Spleen, Stomach, Liver and Gallbladder Diseases, Dongfang Hospital Affiliated to Beijing University of Chinese Medicine, Beijing, China
[8]Department of Acupuncture and Moxibustion, Dongzhimen Hospital Affiliated to Beijing University of Chinese Medicine, Beijing, China

For peer review only - http://bmjopen.bmj.com/site/about/guidelines.xhtml

Acknowledgements

We acknowledge the help and contributions from the nurses, research assistants, acupuncturists, experts and investigators in each cente. We thank the patients with refractory irritable bowel syndrome in this trial.

Authors' contributions

ZGL, HZ and SGY conceived of the study. JZ, MC, and XW are co-first authors. JZ, XW, KY, QSW, YH, JWS, DL and SYL are involved in the conception, design, and critical revision for this study protocol. MC, HZ, XW, SHS, HXL, JFW, XWC and JXN are responsible for recruiting subjects and assessment. ZGL and SGY sought funding and ethical approval and helped with its implementation. HZ is responsible for revising the manuscript. All authors read and approved the final manuscript.

Funding

The National Key Research and Development Program of China (No. 2019YFC1709004)

Competing interests

The authors declare that they have no competing interests.

Patient consent for publication Not required.

Provenance and peer review Not commissioned; externally peer reviewed.

Availability of data and materials

After the study is completed, all relevant data will be shared from the corresponding author on reasonable request.

Open access This is an open access article distributed in accordance with the Creative Commons Attribution Non Commercial (CC BY- NC 4.0) license, which permits others to distribute, remix, adapt, build upon this work non- commercially, and license their derivative works on different terms, provided the original work is properly cited, appropriate credit is given, any changes made indicated, and the use is non-commercial. See: http:// creativecommons. org/ licenses/ by- nc/ 4. 0/.

Figure captions

Figure 1: Trial flow diagram.

Figure 2: Schedule of enrollment, intervention, and assessments of this study protocol.

Figure 3: Locations of acupoints and non-acupoints.

REFERENCES

1 Drossman DA, Hasler WL. Rome IV-functional GI disorders: disorders of gut-brain interaction. *Gastroenterology* 2016;150:1257-61.

For peer review only - http://bmjopen.bmj.com/site/about/guidelines.xhtml

2 Liu J, Hou X. A review of the irritable bowel syndrome investigation on epidemiology, pathogenesis and pathophysiology in China. *J Gastroenterol Hepatol* 2011;26(Suppl 3):88-93.

3 Sperber AD, Dumitrascu D, Fukudo S, *et al.* The global prevalence of IBS in adults remains elusive due to the heterogeneity of studies: a Rome Foundation working team literature review. *Gut* 2017;66:1075-82.

4 Canavan C, West J, Card T. The epidemiology of irritable bowel syndrome. *Clin Epidemiol* 2014;6:71-80.

5 Zhang QE, Wang F, Qin G, *et al.* Depressive symptoms in patients with irritable bowel syndrome: a meta-analysis of comparative studies. *Int J Biol Sci* 2018;14:1504-12.

6 Chen M, Tang TC, Qin D, *et al*. Pharmacologic treatments for irritable bowel syndrome: an umbrella systematic review. *J Gastrointestin Liver Dis* 2020;29:199-209.

7 Spinner HL, Lonardo NW, Mulamalla R, Stehlik J. Ventricular tachycardia associated with high-dose chronic loperamide use. *Pharmacotherapy* 2015;35:234-8.

8 Ford AC, Lacy BE, Harris LA, *et al*. Effect of antidepressants and psychological therapies in irritable bowel syndrome. *Am J Gastroenterol* 2019;114:21-39.

9 Sebastián Sánchez B, Gil Roales-Nieto J, Ferreira NB, *et al*. New psychological therapies for irritable bowel syndrome: mindfulness, acceptance and commitment therapy (ACT). *Rev Esp Enferm Dig* 2017;109:648-57.

10 Dalrymple J, Bullock I. Diagnosis and management of irritable bowel syndrome in adults in primary care: summary of NICE guidance. *BMJ* 2008;336:556-8.

11 Ouyang H, Chen JD. Review article: therapeutic roles of acupuncture in functional gastrointestinal disorders. *Aliment Pharmacol Ther* 2004;20:831-41.

12 Pei L, Geng H, Guo J, *et al*. Effect of acupuncture in patients with irritable bowel syndrome: A randomized controlled trial. *Mayo Clin Proc* 2020;S0025-6196:30151-8.

13 MacPherson H, Tilbrook H, Agbedjro D, *et al*. Acupuncture for irritable bowel syndrome: 2-year follow-up of a randomised controlled trial. *Acupunct Med* 2017;35(1):17-23.

14 MacPherson H, Tilbrook H, Bland JM, *et al*. Acupuncture for irritable bowel syndrome: primary care based pragmatic randomised controlled trial. *BMC Gastroenterol* 2012;12:150.

15 Chan AW, Tetzlaff JM, Altman DG, *et al*. SPIRIT 2013 statement: defining standard protocol items for clinical trials. *Ann Intern Med* 2013;158:200-7.

For peer review only - http://bmjopen.bmj.com/site/about/guidelines.xhtml

16 Pinn DM, Aroniadis OC, Brandt LJ. Is fecal microbiota transplantation the answer for irritable bowel syndrome? A single-center experience. *Am J Gastroenterol* 2014;109:1831-2

17 Moser G, Trägner S, Gajowniczek EE, *et al*. Long-term success of GUT-directed group hypnosis for patients with refractory irritable bowel syndrome: a randomized controlled trial. *Am J Gastroenterol* 2013;108:602-9.

18 Zeevenhooven J, de Bruijn CMA, Vlieger A, *et al*. Protocol for a pilot randomised, double-blind, placebo-controlled trial for assessing the feasibility and efficacy of faecal microbiota transplantation in adolescents with refractory irritable bowel syndrome: FAIS Trial. *BMJ Paediatr Open* 2020;4:e000689.

19 Mearin F, Lacy BE, Chang L, *et al*. Bowel disorders. *Gastroenterology* 2016;S0016-5085:00222-5.

20 Standardization Administration of China: The SAC Register (2006). http://www.sac.gov.cn. Accessed 18 Sept 2006.

21 Hou YQ, Zhang X, Tu JF, *et al*. Efficacy of acupuncture versus sham acupuncture for postprandial distress syndrome: study protocol for a randomized controlled trial. *Trials* 2019;20:65.

22 Francis CY, Morris J, Whorwell PJ. The irritable bowel severity scoring system: a simple method of monitoring irritable bowel syndrome and its progress. *Aliment Pharmacol Ther* 1997;11:395-402.

23 Camilleri M. Editorial: is adequate relief fatally flawed or adequate as an end point in irritable bowel syndrome?. *Am J Gastroenterol* 2009;104:920-2.

24 Martinez AP, de Azevedo GR. The Bristol Stool Form Scale: its translation to Portuguese, cultural adaptation and validation. *Rev Lat Am Enfermagem* 2012;20:583-9.

25 Riegler G, Esposito I. Bristol scale stool form. A still valid help in medical practice and clinical research. *Tech Coloproctol* 2001;53:163-4.

26 Mundt JC, Marks IM, Shear MK, *et al*. The work and social adjustment scale: a simple measure of impairment in functioning. *Br J Psychiatry* 2002;180:461-4.

27 Huang WW, Zhou FS, Bushnell DM, *et al*. Cultural adaptation and application of the IBS-QOL in China: a disease-specific quality-of-life questionnaire. *Qual Life Res* 2007;16:991-6.

28 Cho HS, Park JM, Lim CH, *et al*. Anxiety, depression and quality of life in patients with

For peer review only - http://bmjopen.bmj.com/site/about/guidelines.xhtml

irritable bowel syndrome. *Gut Liver* 2011;5:29-36.

29 Zigmond AS, Snaith RP. The hospital anxiety and depression scale. *Acta Psychiatr Scand* 1983;67:361-70.

30 Zung WW. A rating instrument for anxiety disorders. *Psychosomatics* 1971;12:371-9.

31 Zung WW. A self-rating depression scale. *Arch Gen Psychiatry* 1965;12:63-70.

32 Chow SC, Shao J, Wang H. Sample size calculation in clinical research. *Crc Press* 2003.

33 Buono JL, Carson RT, Flores NM. Health-related quality of life, work productivity, and indirect costs among patients with irritable bowel syndrome with diarrhea. *Health Qual Life Outcomes* 2017;15:35.

34 Masuy I, Pannemans J, Tack J. Irritable bowel syndrome: diagnosis and management. *Minerva Gastroenterol Dietol* 2020;66:136-50.

35 Creed F, Ratcliffe J, Fernandes L, *et al*. Outcome in severe irritable bowel syndrome with and without accompanying depressive, panic and neurasthenic disorders. *Br J Psychiatry* 2005;186:507-15.

36 Emmanuel A, Goosey RW, Wiseman G, *et al*. Impact of symptom severity in patients with diarrhoea-predominant irritable bowel syndrome (IBS-D): results from two separate surveys of HCPs and patients with IBS-D. *BMC Gastroenterol* 2020;20:127.

37 Zheng GZ, Li H, Zhou CL, *et al*. Analysis of regularity of acupuncture and moxibustion in the treatment of D-IBS and C-IBS by data mining. *World Chinese Medicine* 2017;12:684-8.

38 Zheng H, Li Y, Zhang W, *et al*. Electroacupuncture for patients with diarrhea-predominant irritable bowel syndrome or functional diarrhea: A randomized controlled trial. *Medicine (Baltimore)* 2016;95:e3884.

39 Genç F, Tan M. The effect of acupressure application on chemotherapy-induced nausea, vomiting, and anxiety in patients with breast cancer. *Palliat Support Care* 2015;13:275-84.

40 Kazdin AE. Treatment as usual and routine care in research and clinical practice. *Clin Psychol Rev* 2015;42:168-78.

41 Olden KW, Brown AR. Treatment of the severe refractory irritable bowel patient. *Curr Treat Options Gastroenterol* 2006;9:324-30.

42 Creed F, Ratcliffe J, Fernandez L, *et al*. Health-related quality of life and health care costs in severe, refractory irritable bowel syndrome. *Ann Intern Med* 2001;134:860-8.

For peer review only - http://bmjopen.bmj.com/site/about/guidelines.xhtml

43 Everitt HA, Landau S, O'Reilly G, *et al*. Cognitive behavioural therapy for irritable bowel syndrome: 24-month follow-up of participants in the ACTIB randomised trial. *Lancet Gastroenterol Hepatol* 2019;4:863-72.

44 Windgassen S, Moss-Morris R, Everitt H, *et al*. Cognitive and behavioral differences between subtypes in refractory irritable bowel syndrome. *Behav Ther* 2019;50:594-607.

45 Everitt HA, Landau S, O'Reilly G, *et al*. Assessing telephone-delivered cognitive-behavioural therapy (CBT) and web-delivered CBT versus treatment as usual in irritable bowel syndrome (ACTIB): a multicentre randomised trial. *Gut* 2019;68:1613-23.

For peer review only

For peer review only - http://bmjopen.bmj.com/site/about/guidelines.xhtml

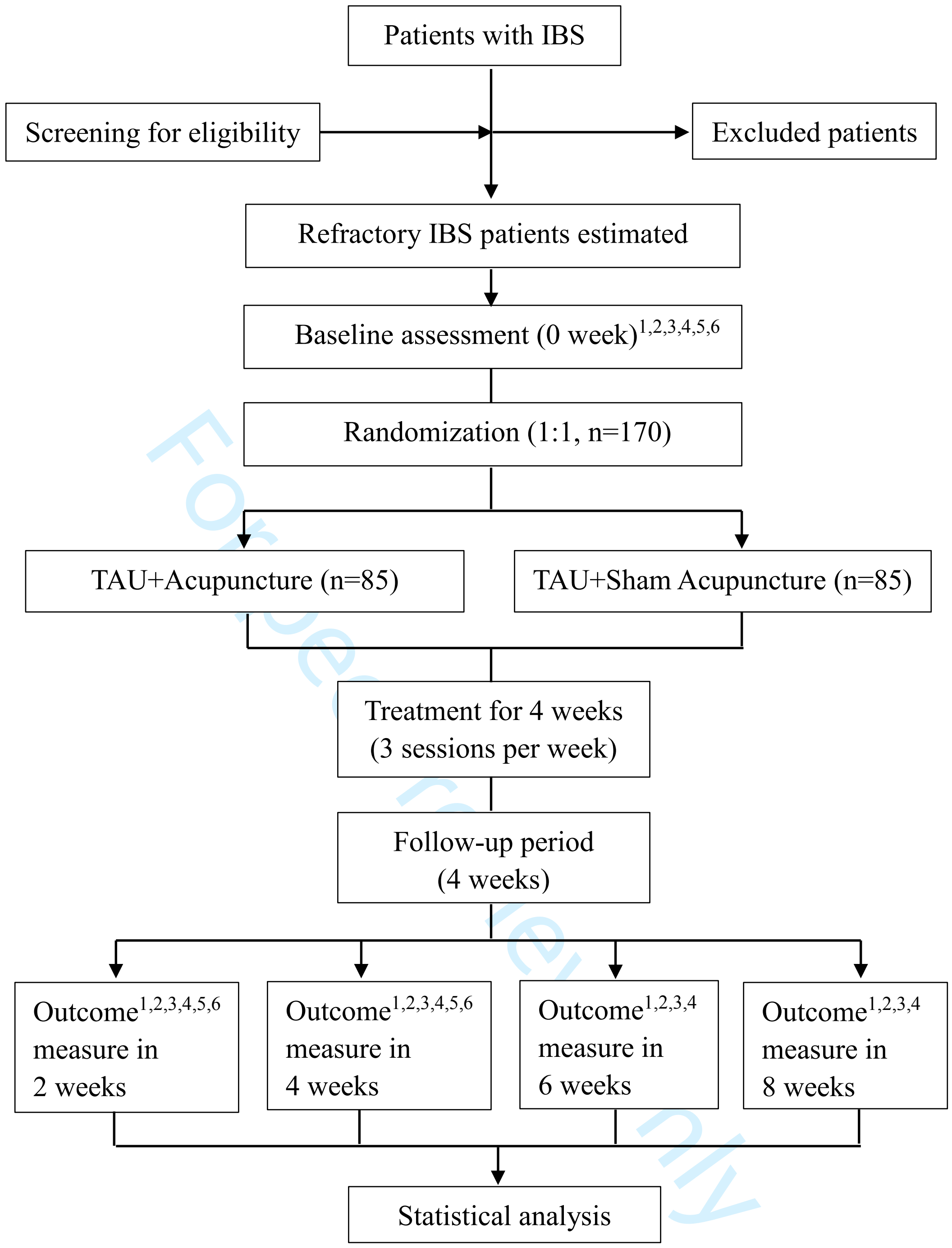

[1] is the IBS symptom severity score
[2] is the times of defecation weekly
[3] is the bristol stool form scale
[4] is the work and social adjustment score
[5] is the IBS-quality of life
[6] are the self-rating depression scale and self-rating anxiety scale anxiety and depression scores
TAU, treatment as usual

Figure 1 Trial flow diagram

For peer review only

	Study Period								
	**Enrollment**	**Allocation**	**Post-allocation**						**Closeout**
**TIME POINT**	w -1	0	AFT	w 1	w 2	w 3	w 4	w 6	w 8
**ENROLLMENT:**									
Eligibility screen	×								
Informed consent	×								
Randomization	×								
Allocation		×							
**INTERVENTIONS:**									
TAU+Acupuncture			◆	—	—	—	◆		
TAU+Sham Acupuncture			◆	—	—	—	◆		
**ASSESSMENTS:**									
IBS symptom severity score		×			×		×	×	×
Adequate relief of global IBS symptoms				×	×	×	×		
Bristol stool form scale		×			×		×	×	×
The frequency of defecation weekly		×			×		×	×	×
Work and social adjustment score		×			×		×	×	×
IBS-quality of life		×			×		×		
The SDS and SAS scores		×			×		×		
Blinding			×				×		
Safety evaluation				×	×	×	×		

Figure 2 Schedule of enrollment, intervention, and assessments of this study protocol. AFT, after the first treatment; SAS, self-rating anxiety scale; SDS, self-rating depression scale; TAU, treatment as usual; w, week.

For peer review only - http://bmjopen.bmj.com/site/about/guidelines.xhtml

For peer review only

For peer review only - http://bmjopen.bmj.com/site/about/guidelines.xhtml

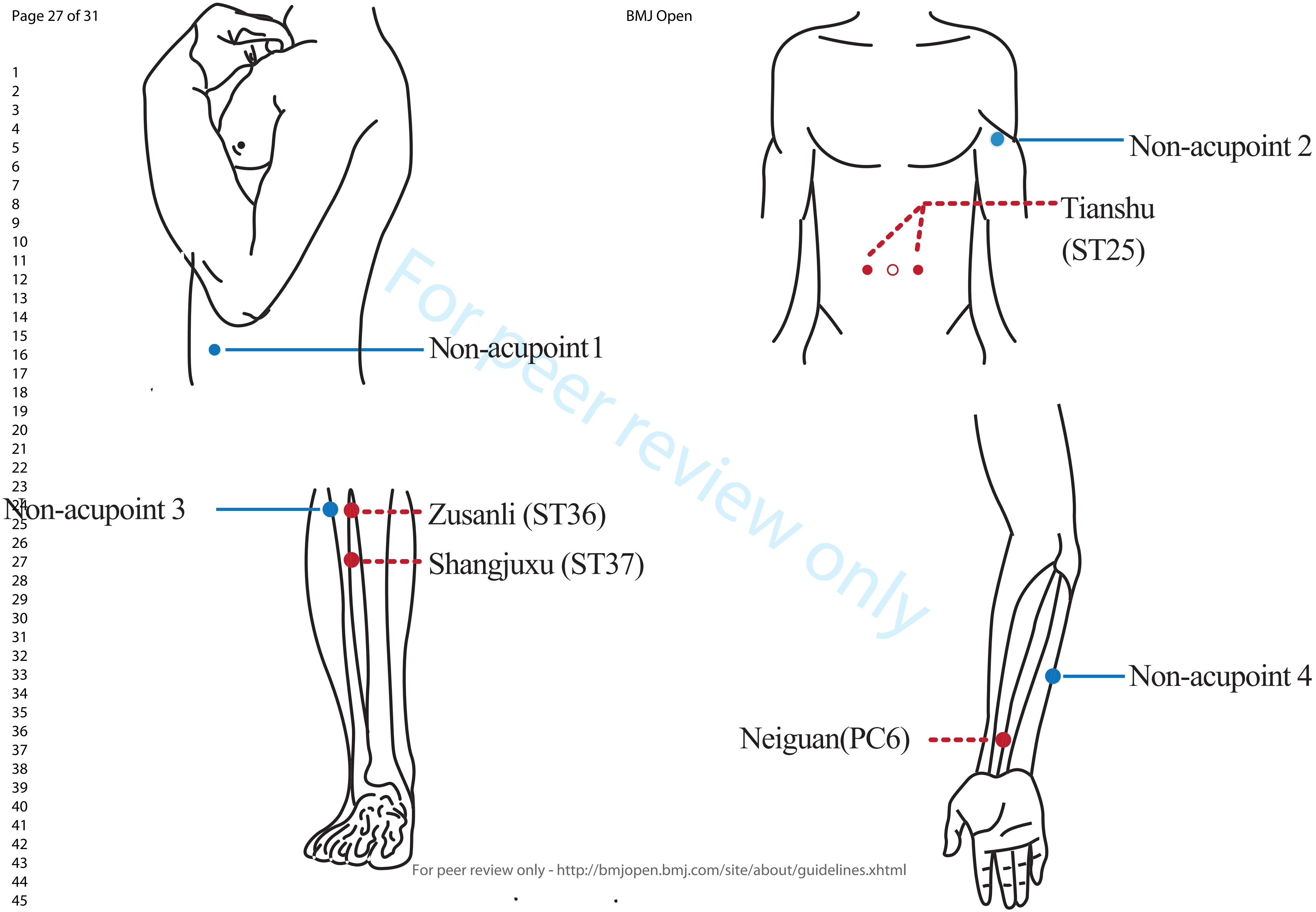

For peer review only

Reporting checklist for protocol of a clinical trial.

Based on the SPIRIT guidelines.

Instructions to authors

Complete this checklist by entering the page numbers from your manuscript where readers will find each of the items listed below.

Your article may not currently address all the items on the checklist. Please modify your text to include the missing information. If you are certain that an item does not apply, please write "n/a" and provide a short explanation.

Upload your completed checklist as an extra file when you submit to a journal.

In your methods section, say that you used the SPIRITreporting guidelines, and cite them as:

Chan A-W, Tetzlaff JM, Gøtzsche PC, Altman DG, Mann H, Berlin J, Dickersin K, Hróbjartsson A, Schulz KF, Parulekar WR, Krleža-Jerić K, Laupacis A, Moher D. SPIRIT 2013 Explanation and Elaboration: Guidance for protocols of clinical trials. BMJ. 2013;346:e7586

		Reporting Item	Page Number
**Administrative information**			
Title	#1	Descriptive title identifying the study design, population, interventions, and, if applicable, trial acronym	1
Trial registration	#2a	Trial identifier and registry name. If not yet registered, name of intended registry	2
Trial registration: data set	#2b	All items from the World Health Organization Trial Registration Data Set	4-16
Protocol version	#3	Date and version identifier	2
Funding	#4	Sources and types of financial, material, and other support	19
Roles and responsibilities: contributorship	#5a	Names, affiliations, and roles of protocol contributors	1,19

For peer review only

Roles and responsibilities: sponsor contact information	#5b	Name and contact information for the trial sponsor	1
Roles and responsibilities: sponsor and funder	#5c	Role of study sponsor and funders, if any, in study design; collection, management, analysis, and interpretation of data; writing of the report; and the decision to submit the report for publication, including whether they will have ultimate authority over any of these activities	19
Roles and responsibilities: committees	#5d	Composition, roles, and responsibilities of the coordinating centre, steering committee, endpoint adjudication committee, data management team, and other individuals or groups overseeing the trial, if applicable (see Item 21a for data monitoring committee)	n/a
**Introduction**			
Background and rationale	#6a	Description of research question and justification for undertaking the trial, including summary of relevant studies (published and unpublished) examining benefits and harms for each intervention	2
Background and rationale: choice of comparators	#6b	Explanation for choice of comparators	2-4
Objectives	#7	Specific objectives or hypotheses	4
Trial design	#8	Description of trial design including type of trial (eg, parallel group, crossover, factorial, single group), allocation ratio, and framework (eg, superiority, equivalence, non-inferiority, exploratory)	4
**Methods: Participants, interventions, and outcomes**			
Study setting	#9	Description of study settings (eg, community clinic, academic hospital) and list of countries where data will be collected. Reference to where list of study sites can be obtained	6
Eligibility criteria	#10	Inclusion and exclusion criteria for participants. If applicable, eligibility criteria for study centres and individuals who will	7

For peer review only

		perform the interventions (eg, surgeons, psychotherapists)	
Interventions: description	#11a	Interventions for each group with sufficient detail to allow replication, including how and when they will be administered	8
Interventions: modifications	#11b	Criteria for discontinuing or modifying allocated interventions for a given trial participant (eg, drug dose change in response to harms, participant request, or improving / worsening disease)	10
Interventions: adherance	#11c	Strategies to improve adherence to intervention protocols, and any procedures for monitoring adherence (eg, drug tablet return; laboratory tests)	10-11
Interventions: concomitant care	#11d	Relevant concomitant care and interventions that are permitted or prohibited during the trial	10-11
Outcomes	#12	Primary, secondary, and other outcomes, including the specific measurement variable (eg, systolic blood pressure), analysis metric (eg, change from baseline, final value, time to event), method of aggregation (eg, median, proportion), and time point for each outcome. Explanation of the clinical relevance of chosen efficacy and harm outcomes is strongly recommended	12-14
Participant timeline	#13	Time schedule of enrolment, interventions (including any run-ins and washouts), assessments, and visits for participants. A schematic diagram is highly recommended (see Figure)	9, Figure2
Sample size	#14	Estimated number of participants needed to achieve study objectives and how it was determined, including clinical and statistical assumptions supporting any sample size calculations	15
Recruitment	#15	Strategies for achieving adequate participant enrolment to reach target sample size	6
**Methods: Assignment of interventions (for controlled trials)**			
Allocation: sequence generation	#16a	Method of generating the allocation sequence (eg, computer-generated random numbers), and list of any factors for stratification. To reduce predictability of a random sequence, details of any planned restriction (eg, blocking) should be provided in a separate document that is unavailable to those who enrol participants or assign interventions	8

For peer review only

Allocation concealment mechanism	#16b	Mechanism of implementing the allocation sequence (eg, central telephone; sequentially numbered, opaque, sealed envelopes), describing any steps to conceal the sequence until interventions are assigned	8
Allocation: implementation	#16c	Who will generate the allocation sequence, who will enrol participants, and who will assign participants to interventions	8
Blinding (masking)	#17a	Who will be blinded after assignment to interventions (eg, trial participants, care providers, outcome assessors, data analysts), and how	8
Blinding (masking): emergency unblinding	#17b	If blinded, circumstances under which unblinding is permissible, and procedure for revealing a participant's allocated intervention during the trial	8
**Methods: Data collection, management, and analysis**			
Data collection plan	#18a	Plans for assessment and collection of outcome, baseline, and other trial data, including any related processes to promote data quality (eg, duplicate measurements, training of assessors) and a description of study instruments (eg, questionnaires, laboratory tests) along with their reliability and validity, if known. Reference to where data collection forms can be found, if not in the protocol	12-14
Data collection plan: retention	#18b	Plans to promote participant retention and complete follow-up, including list of any outcome data to be collected for participants who discontinue or deviate from intervention protocols	12-14
Data management	#19	Plans for data entry, coding, security, and storage, including any related processes to promote data quality (eg, double data entry; range checks for data values). Reference to where details of data management procedures can be found, if not in the protocol	16
Statistics: outcomes	#20a	Statistical methods for analysing primary and secondary outcomes. Reference to where other details of the statistical analysis plan can be found, if not in the protocol	16
Statistics: additional analyses	#20b	Methods for any additional analyses (eg, subgroup and adjusted analyses)	16

For peer review only

Statistics: analysis population and missing data	#20c	Definition of analysis population relating to protocol non-adherence (eg, as randomised analysis), and any statistical methods to handle missing data (eg, multiple imputation)	16
**Methods: Monitoring**			
Data monitoring: formal committee	#21a	Composition of data monitoring committee (DMC); summary of its role and reporting structure; statement of whether it is independent from the sponsor and competing interests; and reference to where further details about its charter can be found, if not in the protocol. Alternatively, an explanation of why a DMC is not needed	16
Data monitoring: interim analysis	#21b	Description of any interim analyses and stopping guidelines, including who will have access to these interim results and make the final decision to terminate the trial	16
Harms	#22	Plans for collecting, assessing, reporting, and managing solicited and spontaneously reported adverse events and other unintended effects of trial interventions or trial conduct	14
Auditing	#23	Frequency and procedures for auditing trial conduct, if any, and whether the process will be independent from investigators and the sponsor	16
**Ethics and dissemination**			
Research ethics approval	#24	Plans for seeking research ethics committee / institutional review board (REC / IRB) approval	16
Protocol amendments	#25	Plans for communicating important protocol modifications (eg, changes to eligibility criteria, outcomes, analyses) to relevant parties (eg, investigators, REC / IRBs, trial participants, trial registries, journals, regulators)	16
Consent or assent	#26a	Who will obtain informed consent or assent from potential trial participants or authorised surrogates, and how (see Item 32)	16
Consent or assent: ancillary studies	#26b	Additional consent provisions for collection and use of participant data and biological specimens in ancillary studies, if applicable	n/a
Confidentiality	#27	How personal information about potential and enrolled participants will be collected, shared, and maintained in order to protect confidentiality before, during, and after the trial	14

For peer review only

Declaration of interests	#28	Financial and other competing interests for principal investigators for the overall trial and each study site	19
Data access	#29	Statement of who will have access to the final trial dataset, and disclosure of contractual agreements that limit such access for investigators	20
Ancillary and post trial care	#30	Provisions, if any, for ancillary and post-trial care, and for compensation to those who suffer harm from trial participation	14
Dissemination policy: trial results	#31a	Plans for investigators and sponsor to communicate trial results to participants, healthcare professionals, the public, and other relevant groups (eg, via publication, reporting in results databases, or other data sharing arrangements), including any publication restrictions	20
Dissemination policy: authorship	#31b	Authorship eligibility guidelines and any intended use of professional writers	n/a
Dissemination policy: reproducible research	#31c	Plans, if any, for granting public access to the full protocol, participant-level dataset, and statistical code	20
**Appendices**			
Informed consent materials	#32	Model consent form and other related documentation given to participants and authorised surrogates	n/a
Biological specimens	#33	Plans for collection, laboratory evaluation, and storage of biological specimens for genetic or molecular analysis in the current trial and for future use in ancillary studies, if applicable	n/a

The SPIRIT checklist is distributed under the terms of the Creative Commons Attribution License CC-BY-ND 3.0. This checklist was completed on 07. October 2020 using https://www.goodreports.org/, a tool made by the EQUATOR Network in collaboration with Penelope.ai

For peer review only

BMJ Open

Efficacy of acupuncture in refractory irritable bowel syndrome: study protocol for a randomised controlled trial

Journal:	*BMJ Open*
Manuscript ID	bmjopen-2020-045655.R1
Article Type:	Protocol
Date Submitted by the Author:	18-May-2021

Complete List of Authors:	Zhao, Jun; Beijing University of Chinese Medicine, School of Acupuncture-Moxibustion and Tuina<br>Chen, Min; Chengdu University of Traditional Chinese Medicine Affiliated Hospital<br>Wang, Xin; Capital Medical University Affiliated Beijing Hospital of Traditional Chinese Medicine<br>Ye, Kun; Chengdu University of Traditional Chinese Medicine, Chengdu, Sichuan, China<br>Shi, Suhua; Department of Rehabilitation, The Third Affiliated Hospital of Beijing University of Chinese Medicine, Beijing, China<br>Li, Huixia; Department of Gastroenterology, Third Affiliated Hospital of Beijing University of Chinese Medicine, Beijing, China<br>Wang, Jianfang; Department of Spleen, Stomach, Liver and Gallbladder Diseases, Dongfang Hospital Affiliated to Beijing University of Chinese Medicine, Beijing, China<br>Chen, Xiaowei; Department of Spleen, Stomach, Liver and Gallbladder Diseases, Dongfang Hospital Affiliated to Beijing University of Chinese Medicine, Beijing, China<br>Ni, Jinxia; Department of Acupuncture and Moxibustion, Dongzhimen Hospital Affiliated to Beijing University of Chinese Medicine, Beijing, China<br>Wei, Qingshuang; School of Acupuncture-Moxibustion and Tuina, Beijing University of Chinese Medicine, Beijing, China<br>Shi, Yunzhou; Acupuncture and Tuina School, Chengdu University of Traditional Chinese Medicine<br>Hu, Yu; School of Acupuncture-Moxibustion and Tuina, Beijing University of Chinese Medicine, Beijing, China<br>Sun, Jingwen; School of Acupuncture-Moxibustion and Tuina, Beijing University of Chinese Medicine, Beijing, China<br>Li, Da; Department of Acupuncture and Moxibustion, Dongzhimen Hospital Affiliated to Beijing University of Chinese Medicine, Beijing, China<br>Liu, Siyuan; School of Acupuncture-Moxibustion and Tuina, Beijing University of Chinese Medicine, Beijing, China<br>Li, Zhigang; Beijing University of Chinese Medicine, School of Acupuncture-moxibustion and Tuina<br>Zheng, Hui ; Chengdu University of Traditional Chinese Medicine,<br>Yu, Shu-guang; Chengdu University of Traditional Chinese Medicine, School of Acupuncture and Tuina

Primary Subject	Complementary medicine

Heading:	
Secondary Subject Heading:	Complementary medicine, Evidence based practice
Keywords:	COMPLEMENTARY MEDICINE, Gastroenterology < INTERNAL MEDICINE, Protocols & guidelines < HEALTH SERVICES ADMINISTRATION & MANAGEMENT

SCHOLARONE™
Manuscripts

I, the Submitting Author has the right to grant and does grant on behalf of all authors of the Work (as defined in the below author licence), an exclusive licence and/or a non-exclusive licence for contributions from authors who are: i) UK Crown employees; ii) where BMJ has agreed a CC-BY licence shall apply, and/or iii) in accordance with the terms applicable for US Federal Government officers or employees acting as part of their official duties; on a worldwide, perpetual, irrevocable, royalty-free basis to BMJ Publishing Group Ltd ("BMJ") its licensees and where the relevant Journal is co-owned by BMJ to the co-owners of the Journal, to publish the Work in this journal and any other BMJ products and to exploit all rights, as set out in our licence.

The Submitting Author accepts and understands that any supply made under these terms is made by BMJ to the Submitting Author unless you are acting as an employee on behalf of your employer or a postgraduate student of an affiliated institution which is paying any applicable article publishing charge ("APC") for Open Access articles. Where the Submitting Author wishes to make the Work available on an Open Access basis (and intends to pay the relevant APC), the terms of reuse of such Open Access shall be governed by a Creative Commons licence – details of these licences and which Creative Commons licence will apply to this Work are set out in our licence referred to above.

Other than as permitted in any relevant BMJ Author's Self Archiving Policies, I confirm this Work has not been accepted for publication elsewhere, is not being considered for publication elsewhere and does not duplicate material already published. I confirm all authors consent to publication of this Work and authorise the granting of this licence.

For peer review only

Efficacy of acupuncture in refractory irritable bowel syndrome: study protocol for a randomised controlled trial

Jun Zhao,[1] Min Chen,[2] Xin Wang,[3] Kun Ye,[4] Suhua Shi,[5] Huixia Li,[6] Jianfang Wang,[7] Xiaowei Chen,[7] Jinxia Ni,[8] Qingshuang Wei,[1] Yunzhou Shi,[4] Yu Hu,[1] Jingwen Sun,[1] Da Li,[1,8] Siyuan Liu,[1] Zhigang Li,[1] Hui Zheng,[4] Shu-Guang Yu[4]

JZ, MC, and XW contributed equally.

Correspondence to

Zhigang Li, School of Acupuncture-Moxibustion and Tuina, Beijing University of Chinese Medicine, Beijing, China (e-mail:lizhigang620@126.com).

Hui Zheng, Chengdu University of Traditional Chinese Medicine, Chengdu, Sichuan, China (e-mail: zhenghui@cdutcm.edu.cn).

Shu-Guang Yu, Chengdu University of Traditional Chinese Medicine, Chengdu, Sichuan, China (e-mail: ysg@cdutcm.edu.cn).

ABSTRACT

Introduction Irritable bowel syndrome (IBS) is a common chronic functional gastrointestinal disorder that presents with abdominal pain/discomfort and altered bowel patterns. IBS has multiple potential causes for which conventional medicines have had limited success, resulting in a significant number of patients who do not sensitively respond to pharmacotherapy for a period of 12 months and who develop a continuing symptom profile (described as refractory IBS) and seek help through (non)pharmacological treatments. The aim of this study is to investigate the efficacy and safety of acupuncture therapy for refractory IBS on the basis of conventional treatments.

Methods and analysis A total of 170 eligible patients who meet the Rome IV criteria for refractory IBS will be randomly allocated to receive acupuncture or sham acupuncture. Each patient will receive 12 sessions of acupuncture over 4 weeks and a 4-week follow-up. The primary outcome will be the IBS symptom severity score (IBS-SSS). Secondary outcomes will include the proportion of participants experiencing adequate relief of global IBS symptoms, the weekly frequency of defecation, the stool properties assessed by the Bristol grading scale, the work and social adjustment scale (WSAS), the IBS-quality of life (IBS-QOL) score, and the self-rating depression scale (SDS) and self-rating anxiety scale (SAS) anxiety and depression scores. Outcome measures will be collected at baseline, 2 weeks and 4 weeks of the intervention, and 6 weeks and 8 weeks after the intervention. Categorical variables will be compared with Fisher's exact test or the Wilcoxon rank-sum test, and continuous variables will be compared using Student's t-test or the Wilcoxon rank-sum test.

Ethics and dissemination The entire project has been approved by the ethics committees of Beijing University of Chinese Medicine (2020BZYLL0507) and Sichuan Province Regional Institution for Conducting Research on Traditional Chinese Medicine (2020KL-025). The outcomes of the trial will be disseminated through peer-reviewed publications.

Trial registration number NCT04276961.

For peer review only - http://bmjopen.bmj.com/site/about/guidelines.xhtml

Strengths and limitations of this study

- This is the first multicentre randomized controlled trial verifying the benefit of acupuncture added to usual treatments for refractory IBS.
- A sham acupuncture group will be used to investigate the placebo effect of acupuncture.
- This trial meets the methodological demand of adequate concealment of randomized group allocations, outcome assessors, and statisticians.
- Participants will be screened for eligible refractory IBS at baseline through three rigorous phases.
- A limitation of this trial is that since a rigorous study design was developed to identify which patients with IBS are "refractory", we must fully consider the differences in the severity of symptoms, the duration of symptoms, the dietary interventions or advice provided, and even the conventional pharmaceutical treatments provided.

INTRODUCTION

Irritable bowel syndrome (IBS) is the most common functional gastrointestinal disorder worldwide.[1] Epidemiological data show that the prevalence of IBS is approximately 5-6% in China,[2] 9.5-9.8% in Asia,[3] and 11% worldwide,[4] which indicates that IBS is a major public health issue. Owing to the complex pathogenesis of IBS, several pathogenic factors (genetic and environmental factors, visceral hypersensitivity, abnormal gut motility, postinfectious inflammatory mechanisms, gut microbiota changes, psychological morbidity, and physical and emotional factors) have been proposed as possible causes.[5] Hence, the study of patients with IBS is still a challenge.

Furthermore, in clinical practice, IBS patients complain about various symptoms, such as changes in bowel habits, abdominal pain, diarrhoea or constipation, and meteorism, which considerably worsen their quality of life (QOL). The Rome IV criteria suggest that IBS treatment is selected on the basis of the symptom type (abdominal pain, diarrhoea, etc.).[1] Simultaneously, patients are stratified into four subtypes: constipation-predominant IBS (IBS-C), diarrhoea-predominant IBS (IBS-D), mixed IBS (IBS-M), and unclassified IBS (IBS-U). Many medications for treating IBS-related symptoms have been developed for various IBS subtypes, including antispasmodics, psychotropic agents, bulking agents, 5-HT receptor antagonists, antibiotics, probiotics and so on. Although 5-HT3 antagonists, 5-HT4 agonists,

For peer review only - http://bmjopen.bmj.com/site/about/guidelines.xhtml

antispasmodics, and alosetron have been shown to be useful for the treatment of IBS;[6] regrettably, some drugs, such as guanylate cyclase-C agonists and loperamide, have been proven to be inadequate for relieving symptoms.[6 7] Similarly, some psychological therapies, such as hypnotherapy, relaxation therapy and cognitive-behavioural therapy, have been proven to be effective treatments for IBS.[8] Even when these available treatments are used, symptoms can persist for more than 12 months in some patients with IBS. These patients with IBS can be considered therapy resistant (refractory).[9 10] Therefore, novel treatment options for IBS, especially refractory IBS, are urgently needed.

Recently, there has been an increase in the use of nonpharmacological therapies, including acupuncture, which is widely accepted as an effective alternative treatment for gastrointestinal disorders in clinical practice.[11] According to reviews of the literature, acupuncture is not only satisfactory for IBS but may also be more effective than pharmacological therapies in alleviating the symptoms and QOL, with its effects lasting up to 12 weeks,[12] even sustaining benefits over a period of 12 months postrandomization.[13 14] However, there are still limitations within previous studies, including small sample sizes, poor quality of the trials and lack of detailed assessments of symptom improvement, such as social activity scales and psychological questionnaires. Meanwhile, few studies have been designed for investigating the treatment of refractory IBS with acupuncture. Therefore, the main objective of this trial is to evaluate, on the basis of conventional treatments, the efficacy of acupuncture versus sham acupuncture for refractory IBS in terms of symptom management.

METHODS/DESIGN

Study design

This multicenter randomized controlled trial will be conducted at 6 hospitals in China: Dongzhimen Hospital Affiliated to Beijing University of Chinese Medicine; Dongfang Hospital Affiliated to Beijing University of Chinese Medicine; Third Hospital Affiliated to Beijing University of Chinese Medicine; Beijing Hospital of Traditional

For peer review only - http://bmjopen.bmj.com/site/about/guidelines.xhtml

Chinese Medicine Affiliated to Capital Medical University; Clinical Medicine College/Teaching Hospital Affiliated to Chengdu University of Traditional Chinese Medicine, and Third Hospital/Acupuncture and Tuina School Affiliated to Chengdu University of Chinese Medicine. The study has been approved by ethics committees and registered on at the www.clinicaltrials.gov on 19 February 2020 (Registration number: NCT04276961). The study protocol will follow the principles of the Declaration of Helsinki and clinical research to Good Clinical Practice (GCP) guidelines, and will be reported based on SPIRIT 2013 Checklist.[15] The checklist is provided in the online supplementary additional file 1.

Eligible patients will be randomly divided into two groups comprising participants who will receive 1 week of wash-out, 12 sessions over 4 weeks of treatment, and 4 weeks of posttreatment follow-up. Outcomes will be assessed at baseline, during the treatment and at the end of the follow-up. A flow diagram of the trial is shown in figure 1.

Patient recruitment

Step 1: Patients meeting the Rome IV IBS criteria (table 1) will be primarily recruited from the outpatient centres of the 6 participating hospitals. The mode of recruitment will also include advertisements through hospital social media sites (WeChat subscription), posters, Sojump, flyers about this trial, outpatient clinics, and publicity at surrounding community service centres. Step 2: Patients 1) with IBS-related symptoms lasting for at least 12 months and 2) without a response to currently available IBS treatments, such as antibiotics or probiotics, dietary changes, antidepressants, or psychotherapies, will be included.[16-18] Clinicians will primarily select patients with suspected refractory IBS for further detailed assessment. Step 3: A clinical research assistant will identify eligible participants for this trial according to the following rigorous inclusion and exclusion criteria (table 2 and table 3). All patients will voluntarily agree to participate and will sign an informed consent document before randomization.

For peer review only - http://bmjopen.bmj.com/site/about/guidelines.xhtml

Table 1 Rome IV criteria: irritable bowel syndrome [19]

Diagnostic time frame	Symptom description	Symptoms
① ≥ 3 months of persistent symptoms with symptom onset at least 6 months before diagnosis<br>② ≥ 1 day per week	Abdominal pain	Recurrent association (2 or more) of the following criteria:<br>①Related to defecation<br>② Associated with a change in the form of stool<br>③ Associated with a change in the frequency of stool

Table 2 Inclusion criteria

Items	
①	Adults aged 18-70 years (either sex);
②	Fulfilment of the Rome IV criteria for IBS;
③	Patients with normal results of tests for occult blood in stool within the past month;
④	Age above 50 years and normal results of colonoscopy within the previous year;
⑤	Presence of symptoms for ≥12 months;
⑥	Absence of response to a minimum of 6 weeks of dietary intervention or advice;
⑦	Absence of response to an adequate dose of at least one conventional pharmacological agent tried for a minimum of 6 weeks;
⑧	Signing of the written informed consent form.

Table 3 Exclusion criteria

Items	
①	Previous colonoscopy, meal barium fluoroscopy, abdominal ultrasound and other examinations revealing severe intestinal organic lesions (including but not limited to ulcerative colitis, familial multiple intestinal polyps, and colorectal cancers);

For peer review only - http://bmjopen.bmj.com/site/about/guidelines.xhtml

② The presence of one or more of the following warning symptoms: unexplained rectal bleeding, a positive faecal occult blood test result; anaemia, abdominal mass, ascites, fever, and emaciation;

③ The presence of other severe medical conditions, such as cardiovascular disease, endocrine disorders, hepatic dysfunction, renal diseases, and cognitive disorders that can affect the outcome assessment results;

④ Unconsciousness, inability to express subjective symptoms of discomfort, and clearly diagnosed severe mental disorders;

⑤ An unstable psychological state or accompanying psychological disorders (SDS>56);

⑥ Present history of pregnancy or lactation;

⑦ Acceptance of acupuncture treatment in the last 3 months;

⑧ Difficulties in attending the trial, such as fear of acupuncture.

Randomization/allocation

A blocked randomization sequence for multiple centres will be generated by an independent professional statistician (Linkermed Tech Co., Ltd) using Statistical Analysis System (SAS) software. All eligible participants will be randomly stratified into the acupuncture group or sham acupuncture group in a 1:1 ratio. Simultaneously, we plan to cooperate with a remote central web-based randomization system. The clinical research assistant will be responsible for enrolling patients, screening patients, obtaining informed consent, requesting randomization and collecting data.

Blinding

Patients as well as research assistants, outcome assessors and statisticians will be blinded to the group assignment. However, due to the nature of acupuncture, the acupuncturists providing the interventions cannot be blinded. To minimize known sources of bias, the number/or location of the acupuncture points and manipulation of needles will be similar in the acupuncture and sham acupuncture groups, thereby

For peer review only - http://bmjopen.bmj.com/site/about/guidelines.xhtml

optimizing the blinding of subjects. Similarly, to avoid the subjective bias of researchers and subjects, the data analysis of the whole experiment will adopt a third-party blind evaluation. The participant's allocated intervention will not be revealed until the statistical analysis reports are finalized.

Interventions

To ensure the safety and proper operation of acupuncture, all acupuncturists will participate in the standard operation process (SOP) of skin disinfection, location of acupoints and nonacupoints, fundamental puncture and needle manipulations before the trials. Treatments will be performed by licenced acupuncturists who have at least 3 years of experience in acupuncture. Acupuncture will be discontinued if the patients experience any adverse events (AEs) after acupuncture. The schedule of enrolment, interventions, assessments, and participant visits is shown in figure 2.

Treatment as usual (TAU)

TAU is defined as the continuation of current therapeutic agents prescribed by a general practitioner or gastroenterologist. All patients will continue TAU during the study period. Any changes in the medications of individual participants will be recorded on diary cards.

Acupuncture group

Patients allocated to the acupuncture group with disposable and sterile acupuncture needles (length: 25-40 mm, diameter: 0.30 mm; Hwatuo, Suzhou, China) will receive treatment with acupuncture three times a week for 4 weeks for a total of 12 treatment sessions. Based on traditional Chinese medicine (TCM) theory and our clinical experience, the prespecified acupoints used here will include bilateral *Tianshu (ST25), Shangjuxu (ST37), Zusanli (ST36), and Neiguan (PC6)* (table 4 and figure 3). The exact location and depth of needling for each point will be determined based on the 2006

For peer review only - http://bmjopen.bmj.com/site/about/guidelines.xhtml

People's Republic of China National Standard: The Name and Location of Acupoints (GB/T 12346-2006).[20] After insertion, manipulations of twirling, lifting, and uniform reinforcing-reducing manipulation will be performed on all needles to elicit "deqi". The compositional sensation of "deqi" includes numbness, soreness, distention, aching, and heaviness, which are believed to be an essential component for acupuncture efficacy.[21] Then, the needles will be removed after a 30-min session with clean cotton balls to prevent bleeding.

Table 4 Locations of acupoints in acupuncture group

Acupoints		Locations
Tianshu (ST25)	Bilateral	On the same level of the umbilicus, and 2 cun* lateral to the anterior midline
Shangjuxu (ST37)	Bilateral	6 cun directly below Dubi (ST35), and one finger-breadth lateral to the anterior border of the tibia
Zusanli (ST36)	Bilateral	3 cun directly below Dubi (ST35), and one finger-breadth lateral to the anterior border of the tibia
Neiguan (PC6)	Bilateral	On the line joining Daling (PC7) and Quze (PC3), between the tendons of palmaris longus and flexor carpi radialis, 2 cun above the transverse crease of the wrist

*1 cun (≈20 mm) is defined as the width of the interphalangeal joint of patient's thumb

Sham acupuncture group

The participants in the sham acupuncture group will also receive the same treatment duration and frequency of sessions as the real acupuncture group. Similarly, sham acupuncture will be performed at predefined bilateral sites (nonacupoints 1-4) not corresponding to acupuncture group traditional points or meridians, penetrating to the same depth but without needle manipulation for "deqi" (table 5 and figure 3). Patients will be informed of the 2 types of acupuncture as follows: "in this trial, different types of acupuncture will be compared, but another approach might not target your additional symptoms." This language will be used to avoid deception while unifying the

For peer review only - http://bmjopen.bmj.com/site/about/guidelines.xhtml

simulation conditions of the two groups as much as possible, mixing real and sham points, and this might enhance patient compliance.

Table 5 Locations of non-acupoints in sham acupuncture group

Non-acupoints		Locations
Non-acupoint 1	Bilateral	In the middle of Daheng (SP15) and Zhangmen (LR13) points (lateral aspect)
Non-acupoint 2	Bilateral	In the medial arm on the anterior border of the insertion of the deltoid muscle at the junction of the deltoid and biceps muscles
Non-acupoint 3	Bilateral	On the same level 1-2 cun outboard lateral of the ST36
Non-acupoint 4	Bilateral	In the middle of the medial epicondyle of the humerus and the styloid process of ulna

*1 cun (≈20 mm) is defined as the width of the interphalangeal joint of patient's thumb

Outcomes

Primary outcome

IBS symptom severity score (IBS-SSS)

According to previous assessment recommendations,[22] the IBS symptom severity score (IBS-SSS) is a validated scoring system that relates to the severity of abdominal pain (IBS-SSS1), frequency of abdominal pain (IBS-SSS2), bloating (IBS-SSS3), satisfaction with bowel habits (IBS-SSS4), and disruption of QOL (IBS-SSS5) and will be used as the primary outcome of this trial. Participants will respond to each question on a 100-point visual analogue scale (VAS). Each of the 5 questions generates a

For peer review only - http://bmjopen.bmj.com/site/about/guidelines.xhtml

maximum score of 100 points, and the total is scored from 0 to 500, with higher scores indicating severe symptoms: normal (< 75), mild IBS (75-175), moderate IBS (175-300), and severe IBS (>300). IBS-SSS will be assessed at baseline and 2, 4, 6 and 8 weeks after randomization.

Secondary outcomes

Adequate relief of global IBS symptoms

During our treatment period, patients will respond weekly to the question: "In regard to all your symptoms of IBS, as compared with the way you felt before you added acupuncture therapy on the basis of conventional treatments, have you, in the past 7 days, had adequate relief of your IBS symptoms?" (yes/no). "Yes" will be regarded as a positive response to treatment.[23]

Bristol Stool Form Scale

The Bristol Stool Form Scale classifies stool consistency into 7 types encompassing complications from constipation to diarrhoea.[24] In this trial, participants will be asked to record the weekly defecation types and scores. The scores obtained from the Bristol scale will be assessed at baseline and weeks 2, 4, 6 and 8.

The weekly frequency of defecation

The frequency of bowel movements during the week before each visit will be assessed at baseline and 2, 4, 6 and 8 weeks after randomization. A normal defecation is defined as a daily stool frequency ≤ 3 times and a stool consistency of type 4 (like a sausage or snake; smooth and soft).[25]

Work and Social Adjustment Scale (WSAS)

The WSAS will be assessed at baseline and weeks 2, 4, 6 and 8 using a 5-domain scale with a total possible score of 40, with each item scoring from 0 (not affected) to 8 (severely affected). The scale is used to measure problems in functioning with work, home management, social activities, private leisure activities, and family and

For peer review only - http://bmjopen.bmj.com/site/about/guidelines.xhtml

relationships.[26]

IBS-Quality of Life (IBS-QOL)

The IBS-QOL questionnaire consists of 34 items; each item includes a 5-point response scale and is a valid and reliable instrument to establish changes in health-related QOL of patients with IBS.[27] Patients themselves will be required to complete the questionnaire at baseline and weeks 2 and 4 after the treatment. Then, the total scores of each item will be calculated and analysed, with higher scores suggestive of better QOL.

Self-Rating Anxiety Scale (SAS) and Self-Rating Depression Scale (SDS)

The majority of IBS patients have comorbid anxiety and/or depression, which are in turn strongly associated with symptom severity.[28 29] Hence, anxiety and depression symptoms will be assessed at baseline and weeks 2 and 4 using the SAS and SDS. It is noteworthy that SDS>56 will be excluded before the first treatment because of an unstable psychological state or accompanying psychological disorders. According to statistical standards, the total score is multiplied by 1.25 and is then converted into a standardized score ranging from 25 to 100. For SAS, grades 1, 2 and 3 are related to scores of 53-62, 63-72 and >72, respectively.[30] For SDS, grades 1, 2 and 3 correspond to scores of 50-60, 61-70 and >70, respectively.[31] Higher scores indicate more severe anxiety and depression.

Blinding assessment

To test the patient-blinding effects, all patients will be asked to guess whether they have received acupuncture or sham acupuncture at the end of the first treatment session and the twelfth treatment session.

Safety evaluation

In this trial, AEs will be monitored during the 4 weeks of treatment and the next 4 weeks of follow-up. Any adverse reactions that occurred during the study will be recorded.

For peer review only - http://bmjopen.bmj.com/site/about/guidelines.xhtml

Common side effects of acupuncture or sham acupuncture include (but are not limited to) subcutaneous haematoma, local bleeding, skin bruising, needle site itching, continuous needle site pain, muscle spasm, and dizziness. In the event of a serious adverse event (SAE), the necessary measures will be taken immediately for the safety of the subject. The time of occurrence, severity, duration, outcome, and treatment-related status will be reported to the Medical Ethics Committee within 24 h.

Sample size and statistical analysis

Based on two sample size mean literature data speculation and previous clinical experience,[12 32] we anticipate the scores of two acupuncture groups on the continuation of current medications as follows: responders can improve refractory IBS on the IBS-SSS with scores of 30 and 15, respectively. The standard deviation is set at 15, and the ratio between the acupuncture and sham acupuncture groups is 1:1. The sample size is calculated with a 20% type II error rate (80% power) and 5% type I error. The superiority trial will be adopted to minimize the difference between the two groups, and a 10-point is expected. In total, a sample size of 154 (77 patients in each group) is estimated. Allowing for 10% loss to follow-up, the sample size expands to 85 patients in each group.

All analyses will be performed using the intent-to-treat (ITT) and the per-protocol (PP) populations. ITT requires all participants to receive a baseline assessment of the primary outcome and at least 1 acupuncture session or 1 sham acupuncture administration. Missing values will be imputed through the multiple imputation method. The PP population is usually defined as patients who complete at least 80% of the treatment protocol without major protocol violations. The primary outcome will be assessed using analysis of covariance (ANCOVA) and adjusted for baseline total IBS-SSS. For other secondary outcomes: continuous variables will be analysed using Student's t-test or the Wilcoxon rank-sum test, and data will be represented as the mean ± SD or median; categorical variables will be calculated using Fisher's exact test or the Wilcoxon rank-sum test, and data will be presented as frequencies (percentages). In terms of efficacy factors, other covariates, such as age, sex, disease duration,

For peer review only - http://bmjopen.bmj.com/site/about/guidelines.xhtml

conventional pharmacological agent classification and so on, will be considered for further revision. The differences in blinding and AEs between the groups will be compared using the chi-square test. Moreover, we have decided to carry out a subgroup analysis based on patients with IBS subtypes, avoiding a heterogeneous patient population that may affect the efficacy results.

Statistical analysis of all data in this study will be performed by a specialized third-party statistician evaluation. SAS, version 8.2 statistical software (SAS Institute, Inc.) will be used for data analysis. A two-sided $P<0.05$ will be considered statistically significant.

Data management and quality control

To guarantee the objectivity of the data, individual participant data will not be disclosed outside. All researchers will receive training regarding data management. Data entry and management will be conducted using a database software clinical trial data management system by data administrators of the China Academy of Chinese Medical Science. An electronic case report form (eCRF) will be designed to collect all clinical observation results of each participant before recruitment. The research assistant is responsible for verifying the accuracy and integrity of data to prevent any detection of errors, omissions, or items requiring changes or clarification. Data lockup will be implemented by the data administrators on completion of the study. All paper files and electronic data will be kept for 5 years after publication and destroyed thereafter.

To guarantee the quality of this trial, a prespecified SOP, including recruitment and screening of participants, randomization, laboratory detection results, intervention, details in filling the case report form (CRF), assessment of outcomes, data management, and AEs of acupuncture, will be unified and trained through a multicentre internal study for consistency of results. Moreover, we will set up a Data and Safety Monitoring Board, an independent advisory group, to review and interpret data generated from the study, and a meeting will be held every 3 months to report the research progress. Each amendment of the protocol will conform to good clinical practice (GCP) principles and will maintain the ethical standards for randomized controlled trials (RCTs).

For peer review only - http://bmjopen.bmj.com/site/about/guidelines.xhtml

Patients and public involvement

Patients in this trial will not be involved in the design or conduct of the study or the outcome assessments. However, we are planning to disseminate our research to the participants and the public, through means such as publicising our research on hospital social media sites (WeChat subscription) and various academic lectures.

Ethics and dissemination

This study conforms to the principles of the Declaration of Helsinki and relevant ethical guidelines. The entire project has been approved by the ethics committees of Beijing University of Chinese Medicine (ID: 2020BZYLL0507) and Sichuan Province Regional Institution for Conducting Research on Traditional Chinese Medicine (ID: 2020KL-025). Written informed consent will be obtained from patients prior to enrolment in the study. The outcomes of the trial will be disseminated through peer-reviewed publications.

Discussion

IBS not only impacts patients' health-related QOL, resulting in a large economic burden on healthcare systems, but also impairs work productivity and individuals' ability to perform daily activities, increasing employers' indirect costs.[33] The currently available pharmacological treatments still have some limitations, resulting in many refractory IBS patients seeking help through complementary and alternative medicine.[34]

However, when considering refractory IBS treatment, the symptoms should be highly addressed to maintain optimal results and enhance the QOL of individuals. Patients with IBS, especially refractory IBS, have more severe psychosomatic disorders.[35] Patients who are classified as having severe IBS agree with the statement: 'When my IBS is bad, I wish I was dead'.[36] Hence, another important treatment principle for refractory IBS is improving mental/emotional problems. From a TCM perspective, acupoint selection is a key factor in the success of acupuncture in treating

For peer review only - http://bmjopen.bmj.com/site/about/guidelines.xhtml

diseases. According to previous studies and our previous reviews, the basic acupuncture points important for relieving diarrhoea symptoms are ST25, ST36 and ST37.[37 38] However, acupuncture treatment for psychosomatic diseases highlights the specific functions of acupoints. Clinical evidence has shown that PC6 can improve anxiety symptoms well.[39] Hence, the ST25, ST36, ST37 and PC6 points included in this trial are selected in accordance with not only the TCM treatment principles of "syndrome differentiation" but also clinical practice guidelines in China. However, for these points, scientific evidence is sparse, and high-quality research is often lacking, leading to inconclusive results.[34] As such, a multicentre RCT with a large sample size is urgently needed to evaluate the efficacy of acupuncture in improving refractory IBS symptoms within the evidence-based medical framework.

In view of the complex psychological feelings of refractory patients, acupuncture treatment evaluations will be conducted in clinical settings in which usual treatment is routinely carried out. The many benefits of TAU include surmounting critical ethical issues (e.g., withholding treatment)[40] while controlling for many common factors (e.g., psychological concerns, dietary factors, and medication changes) that can contribute to change. Moreover, because it is uncertain whether the long-term clinical beneficial effect of acupuncture on refractory IBS is sustained, another possible advantage of TAU is that research staff may establish good communication relationships with patients, thus avoiding potential dropouts. In this way, we can cautiously extrapolate the long-term effect of acupuncture.

Nevertheless, this study still has several limitations that should be noted. First, identifying which patients with IBS are "refractory" is challenging because of the differences in the severity of symptoms, usual treatments administered and duration of symptoms.[41] On the basis of a literature review, refractory IBS patients were defined as those who had an average daily pain rate ≥30 mm on the pain component scale of the IBS-SSS,[22] who failed to respond to usual medical treatment (education, dietary advice, antispasmodic agents, antidiarrhoeal medication, antidepressants, fibre-based medications, etc.),[16-18 42 43] and who had IBS symptoms for 12 months or more.[9 10 43-45] Second, to ensure a reliable sample of refractory IBS patients, we plan to conduct two

For peer review only - http://bmjopen.bmj.com/site/about/guidelines.xhtml

rounds of screening: In the first step, the clinicians will primarily select patients with suspected refractory IBS according to the above literature review items. Then, a clinical research assistant will recommend the eligible participants for this trial according to the following improved critical inclusion criteria: 1) there is an absence of response to an adequate dose of at least one conventional pharmacological agent tried for a minimum of 6 weeks; and 2) there is an absence of response to a minimum of 6 weeks of dietary intervention or advice. Moreover, we attach great importance to the potential risk of symptoms in refractory patients. Patients with one or more of the following warning symptoms will be excluded: unexplained rectal bleeding, a positive faecal occult blood test result; anaemia, abdominal mass, ascites, fever, and emaciation. All participants will have to fill in the "Filter table" in advance to guarantee that all inclusion criteria will be met.

We hope that at the end of this trial, the results will provide more reliable evidence and reveal the value of clinical acupoint selection guidelines in the treatment of refractory IBS with acupuncture.

TRIAL STATUS

This trial is currently in the preparation phase. No patient involved.

Author affiliations

[1]School of Acupuncture-Moxibustion and Tuina, Beijing University of Chinese Medicine, Beijing, China

[2]Colorectal Department/ Clinical Medicine College, Hospital of Chengdu University of Traditional Chinese Medicine, Chengdu, China

[3]Department of Acupuncture and Moxibustion, Beijing Hospital of Traditional Chinese Medicine, Capital Medical University, Beijing, China

[4]Chengdu University of Traditional Chinese Medicine, Chengdu, Sichuan, China

[5]Department of Rehabilitation, Third Affiliated Hospital of Beijing University of Chinese Medicine, Beijing, China

[6]Department of Gastroenterology, Third Affiliated Hospital of Beijing University of Chinese Medicine, Beijing, China

[7]Department of Spleen, Stomach, Liver and Gallbladder Diseases, Dongfang Hospital Affiliated to Beijing University of Chinese Medicine, Beijing, China

[8]Department of Acupuncture and Moxibustion, Dongzhimen Hospital Affiliated to Beijing University of Chinese Medicine, Beijing, China

For peer review only - http://bmjopen.bmj.com/site/about/guidelines.xhtml

Acknowledgements

We acknowledge the help and contributions from the nurses, research assistants, acupuncturists, experts and investigators in each centre. We thank the patients with refractory irritable bowel syndrome in this trial.

Authors' contributions

ZGL, HZ and SGY conceived of the study. JZ, MC, and XW are co-first authors. JZ, XW, KY, QSW, YH, YZS, JWS, DL and SYL are involved in the conception, design, and critical revision for this study protocol. MC, HZ, XW, SHS, HXL, JFW, XWC and JXN are responsible for recruiting subjects and assessment. ZGL and SGY sought funding and ethical approval and helped with its implementation. HZ is responsible for revising the manuscript. All authors read and approved the final manuscript.

Funding

The National Key Research and Development Program of China (No. 2019YFC1709004)

Competing interests

The authors declare that they have no competing interests.

Patient consent for publication Not required.

Provenance and peer review Not commissioned; externally peer reviewed.

Availability of data and materials

After the study is completed, all relevant data will be shared from the corresponding author on reasonable request.

Open access This is an open access article distributed in accordance with the Creative Commons Attribution Non Commercial (CC BY- NC 4.0) license, which permits others to distribute, remix, adapt, build upon this work non- commercially, and license their derivative works on different terms, provided the original work is properly cited, appropriate credit is given, any changes made indicated, and the use is non-commercial. See: http://creativecommons. org/licenses/by-nc/4.0/.

Figure captions

Figure 1: Trial flow diagram.

Figure 2: Schedule of enrollment, intervention, and assessments of this study protocol.

Figure 3: Locations of acupoints and non-acupoints.

REFERENCES

1 Drossman DA, Hasler WL. Rome IV-functional GI disorders: disorders of gut-brain interaction. *Gastroenterology* 2016;150:1257-61.

2 Liu J, Hou X. A review of the irritable bowel syndrome investigation on epidemiology,

pathogenesis and pathophysiology in China. *J Gastroenterol Hepatol* 2011;26(Suppl 3):88-93.

3 Sperber AD, Dumitrascu D, Fukudo S, *et al.* The global prevalence of IBS in adults remains elusive due to the heterogeneity of studies: a Rome Foundation working team literature review. *Gut* 2017;66:1075-82.

4 Canavan C, West J, Card T. The epidemiology of irritable bowel syndrome. *Clin Epidemiol* 2014;6:71-80.

5 Zhang QE, Wang F, Qin G, *et al.* Depressive symptoms in patients with irritable bowel syndrome: a meta-analysis of comparative studies. *Int J Biol Sci* 2018;14:1504-12.

6 Chen M, Tang TC, Qin D, *et al*. Pharmacologic treatments for irritable bowel syndrome: an umbrella systematic review. *J Gastrointestin Liver Dis* 2020;29:199-209.

7 Spinner HL, Lonardo NW, Mulamalla R, Stehlik J. Ventricular tachycardia associated with high-dose chronic loperamide use. *Pharmacotherapy* 2015;35:234-8.

8 Ford AC, Lacy BE, Harris LA, *et al*. Effect of antidepressants and psychological therapies in irritable bowel syndrome. *Am J Gastroenterol* 2019;114:21-39.

9 Sebastián Sánchez B, Gil Roales-Nieto J, Ferreira NB, *et al*. New psychological therapies for irritable bowel syndrome: mindfulness, acceptance and commitment therapy (ACT). *Rev Esp Enferm Dig* 2017;109:648-57.

10 Dalrymple J, Bullock I. Diagnosis and management of irritable bowel syndrome in adults in primary care: summary of NICE guidance. *BMJ* 2008;336:556-8.

11 Ouyang H, Chen JD. Review article: therapeutic roles of acupuncture in functional gastrointestinal disorders. *Aliment Pharmacol Ther* 2004;20:831-41.

12 Pei L, Geng H, Guo J, *et al*. Effect of acupuncture in patients with irritable bowel syndrome: A randomized controlled trial. *Mayo Clin Proc* 2020;S0025-6196:30151-8.

13 MacPherson H, Tilbrook H, Agbedjro D, *et al*. Acupuncture for irritable bowel syndrome: 2-year follow-up of a randomised controlled trial. *Acupunct Med* 2017;35(1):17-23.

14 MacPherson H, Tilbrook H, Bland JM, *et al*. Acupuncture for irritable bowel syndrome: primary care based pragmatic randomised controlled trial. *BMC Gastroenterol* 2012;12:150.

15 Chan AW, Tetzlaff JM, Altman DG, *et al*. SPIRIT 2013 statement: defining standard protocol items for clinical trials. *Ann Intern Med* 2013;158:200-7.

16 Pinn DM, Aroniadis OC, Brandt LJ. Is fecal microbiota transplantation the answer for irritable

For peer review only - http://bmjopen.bmj.com/site/about/guidelines.xhtml

bowel syndrome? A single-center experience. *Am J Gastroenterol* 2014;109:1831-2

17 Moser G, Trägner S, Gajowniczek EE, *et al*. Long-term success of GUT-directed group hypnosis for patients with refractory irritable bowel syndrome: a randomized controlled trial. *Am J Gastroenterol* 2013;108:602-9.

18 Zeevenhooven J, de Bruijn CMA, Vlieger A, *et al*. Protocol for a pilot randomised, double-blind, placebo-controlled trial for assessing the feasibility and efficacy of faecal microbiota transplantation in adolescents with refractory irritable bowel syndrome: FAIS Trial. *BMJ Paediatr Open* 2020;4:e000689.

19 Mearin F, Lacy BE, Chang L, *et al*. Bowel disorders. *Gastroenterology* 2016;S0016-5085:00222-5.

20 Standardization Administration of China: The SAC Register (2006). http://www.sac.gov.cn. Accessed 18 Sept 2006.

21 Hou YQ, Zhang X, Tu JF, *et al*. Efficacy of acupuncture versus sham acupuncture for postprandial distress syndrome: study protocol for a randomized controlled trial. *Trials* 2019;20:65.

22 Francis CY, Morris J, Whorwell PJ. The irritable bowel severity scoring system: a simple method of monitoring irritable bowel syndrome and its progress. *Aliment Pharmacol Ther* 1997;11:395-402.

23 Camilleri M. Editorial: is adequate relief fatally flawed or adequate as an end point in irritable bowel syndrome?. *Am J Gastroenterol* 2009;104:920-2.

24 Martinez AP, de Azevedo GR. The Bristol Stool Form Scale: its translation to Portuguese, cultural adaptation and validation. *Rev Lat Am Enfermagem* 2012;20:583-9.

25 Riegler G, Esposito I. Bristol scale stool form. A still valid help in medical practice and clinical research. *Tech Coloproctol* 2001;53:163-4.

26 Mundt JC, Marks IM, Shear MK, *et al*. The work and social adjustment scale: a simple measure of impairment in functioning. *Br J Psychiatry* 2002;180:461-4.

27 Huang WW, Zhou FS, Bushnell DM, *et al*. Cultural adaptation and application of the IBS-QOL in China: a disease-specific quality-of-life questionnaire. *Qual Life Res* 2007;16:991-6.

28 Cho HS, Park JM, Lim CH, *et al*. Anxiety, depression and quality of life in patients with irritable bowel syndrome. *Gut Liver* 2011;5:29-36.

For peer review only - http://bmjopen.bmj.com/site/about/guidelines.xhtml

29 Zigmond AS, Snaith RP. The hospital anxiety and depression scale. *Acta Psychiatr Scand* 1983;67:361-70.

30 Zung WW. A rating instrument for anxiety disorders. *Psychosomatics* 1971;12:371-9.

31 Zung WW. A self-rating depression scale. *Arch Gen Psychiatry* 1965;12:63-70.

32 Chow SC, Shao J, Wang H. Sample size calculation in clinical research. *Crc Press* 2003.

33 Buono JL, Carson RT, Flores NM. Health-related quality of life, work productivity, and indirect costs among patients with irritable bowel syndrome with diarrhea. *Health Qual Life Outcomes* 2017;15:35.

34 Masuy I, Pannemans J, Tack J. Irritable bowel syndrome: diagnosis and management. *Minerva Gastroenterol Dietol* 2020;66:136-50.

35 Creed F, Ratcliffe J, Fernandes L, *et al*. Outcome in severe irritable bowel syndrome with and without accompanying depressive, panic and neurasthenic disorders. *Br J Psychiatry* 2005;186:507-15.

36 Emmanuel A, Goosey RW, Wiseman G, *et al*. Impact of symptom severity in patients with diarrhoea-predominant irritable bowel syndrome (IBS-D): results from two separate surveys of HCPs and patients with IBS-D. *BMC Gastroenterol* 2020;20:127.

37 Zheng GZ, Li H, Zhou CL, *et al*. Analysis of regularity of acupuncture and moxibustion in the treatment of D-IBS and C-IBS by data mining. *World Chinese Medicine* 2017;12:684-8.

38 Zheng H, Li Y, Zhang W, *et al*. Electroacupuncture for patients with diarrhea-predominant irritable bowel syndrome or functional diarrhea: A randomized controlled trial. *Medicine (Baltimore)* 2016;95:e3884.

39 Genç F, Tan M. The effect of acupressure application on chemotherapy-induced nausea, vomiting, and anxiety in patients with breast cancer. *Palliat Support Care* 2015;13:275-84.

40 Kazdin AE. Treatment as usual and routine care in research and clinical practice. *Clin Psychol Rev* 2015;42:168-78.

41 Olden KW, Brown AR. Treatment of the severe refractory irritable bowel patient. *Curr Treat Options Gastroenterol* 2006;9:324-30.

42 Creed F, Ratcliffe J, Fernandez L, *et al*. Health-related quality of life and health care costs in severe, refractory irritable bowel syndrome. *Ann Intern Med* 2001;134:860-8.

43 Everitt HA, Landau S, O'Reilly G, *et al*. Cognitive behavioural therapy for irritable bowel

For peer review only

For peer review only - http://bmjopen.bmj.com/site/about/guidelines.xhtml

syndrome: 24-month follow-up of participants in the ACTIB randomised trial. *Lancet Gastroenterol Hepatol* 2019;4:863-72.

44 Windgassen S, Moss-Morris R, Everitt H, *et al*. Cognitive and behavioral differences between subtypes in refractory irritable bowel syndrome. *Behav Ther* 2019;50:594-607.

45 Everitt HA, Landau S, O'Reilly G, *et al*. Assessing telephone-delivered cognitive-behavioural therapy (CBT) and web-delivered CBT versus treatment as usual in irritable bowel syndrome (ACTIB): a multicentre randomised trial. *Gut* 2019;68:1613-23.

For peer review only

For peer review only - http://bmjopen.bmj.com/site/about/guidelines.xhtml

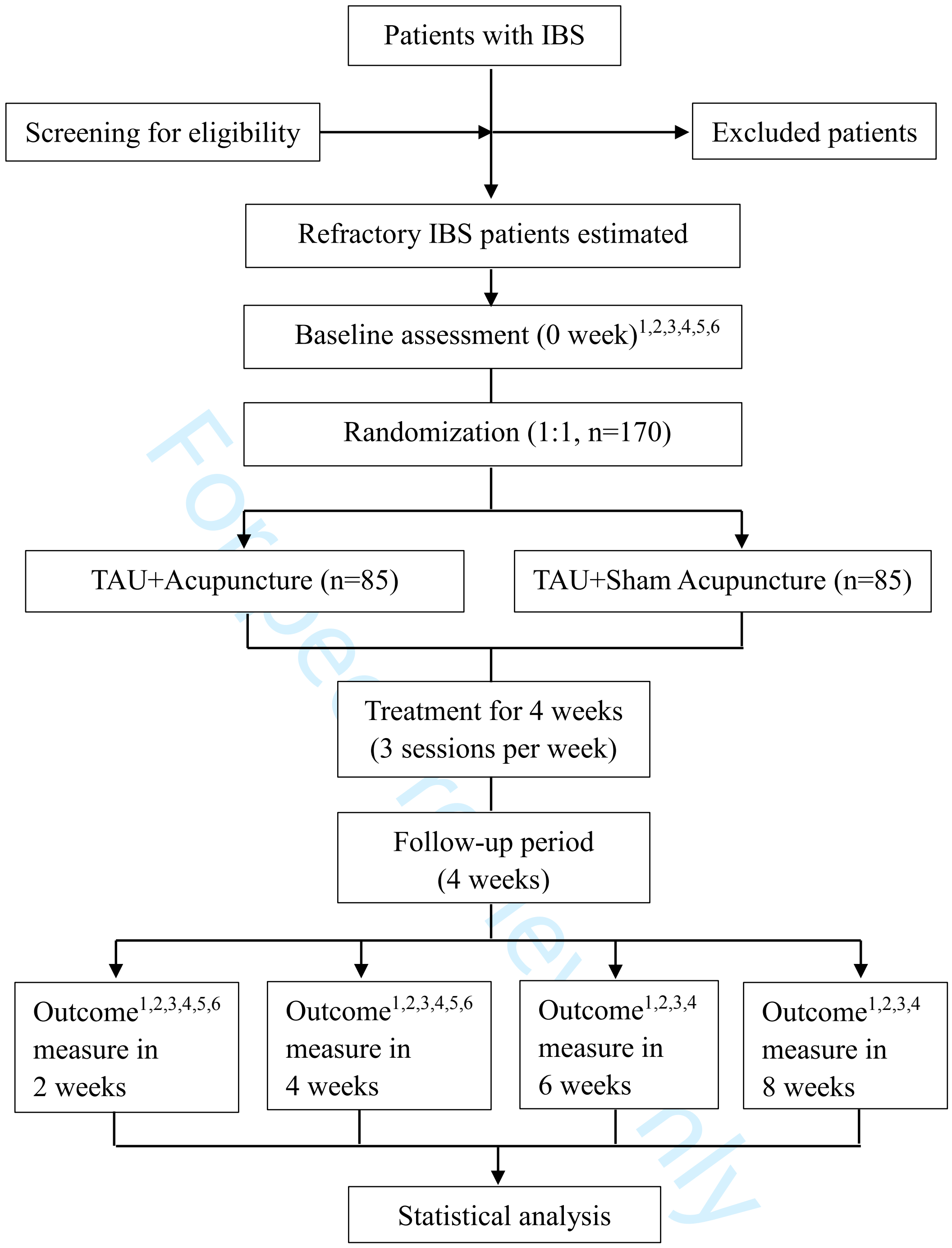

[1] is the IBS symptom severity score
[2] is the times of defecation weekly
[3] is the bristol stool form scale
[4] is the work and social adjustment score
[5] is the IBS-quality of life
[6] are the self-rating depression scale and self-rating anxiety scale anxiety and depression scores
TAU, treatment as usual

Figure 1 Trial flow diagram

For peer review only - http://bmjopen.bmj.com/site/about/guidelines.xhtml

	Study Period								
	**Enrollment**	**Allocation**	**Post-allocation**						**Closeout**
**TIME POINT**	w -1	0	AFT	w 1	w 2	w 3	w 4	w 6	w 8
**ENROLLMENT:**									
Eligibility screen	×								
Informed consent	×								
Randomization	×								
Allocation		×							
**INTERVENTIONS:**									
TAU+Acupuncture			◆——	————	————	————	——◆		
TAU+Sham Acupuncture			◆——	————	————	————	——◆		
**ASSESSMENTS:**									
IBS symptom severity score		×			×		×	×	×
Adequate relief of global IBS symptoms				×	×	×	×		
Bristol stool form scale		×			×		×	×	×
The frequency of defecation weekly		×			×		×	×	×
Work and social adjustment score		×			×		×	×	×
IBS-quality of life		×			×		×		
The SDS and SAS scores		×			×		×		
Blinding			×				×		
Safety evaluation				×	×	×	×		

For peer review only

Figure 2 Schedule of enrollment, intervention, and assessments of this study protocol. AFT, after the first treatment; SAS, self-rating anxiety scale; SDS, self-rating depression scale; TAU, treatment as usual; w, week.

For peer review only - http://bmjopen.bmj.com/site/about/guidelines.xhtml

For peer review only

For peer review only - http://bmjopen.bmj.com/site/about/guidelines.xhtml

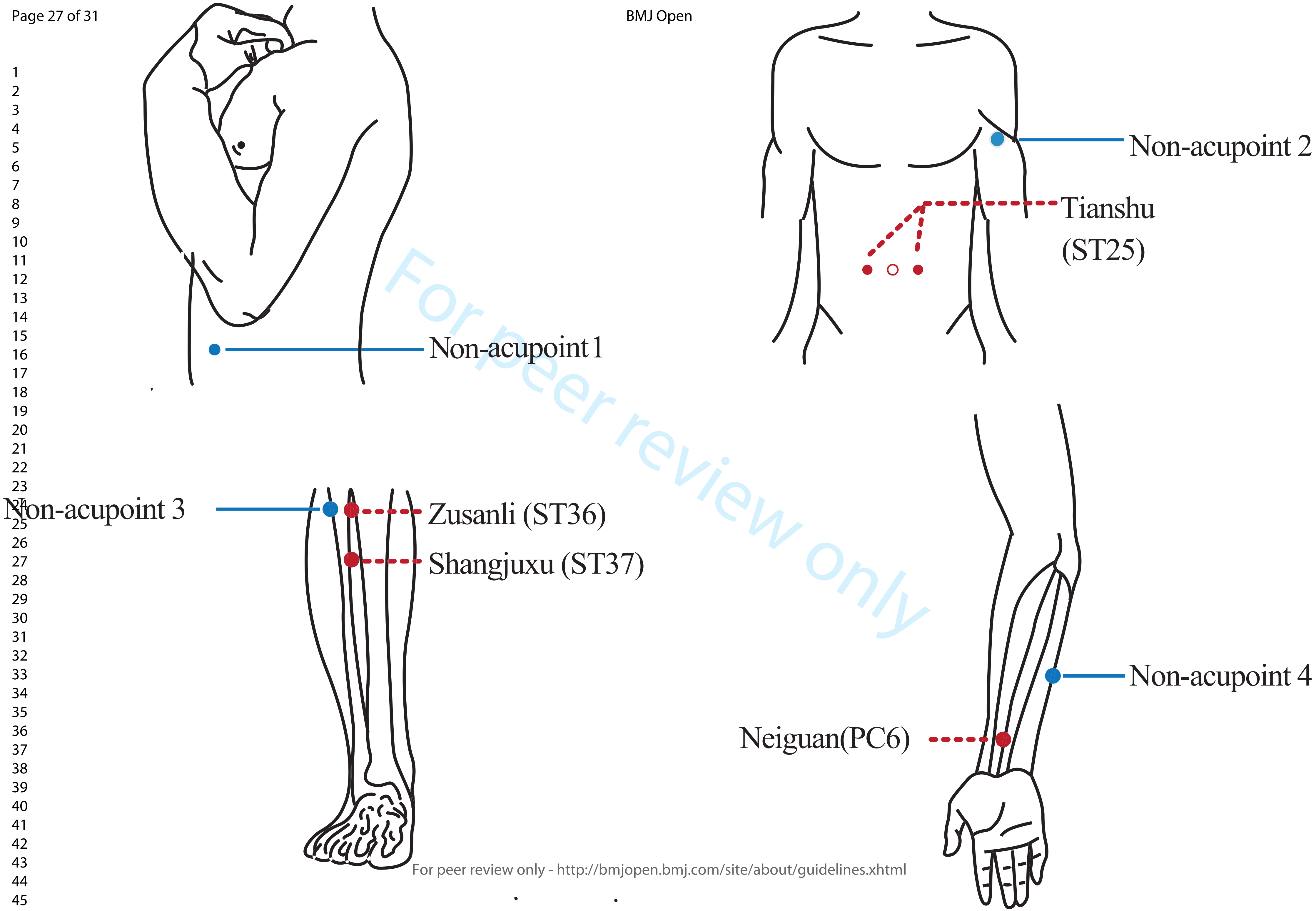

For peer review only - http://bmjopen.bmj.com/site/about/guidelines.xhtml

Reporting checklist for protocol of a clinical trial.

Based on the SPIRIT guidelines.

Instructions to authors

Complete this checklist by entering the page numbers from your manuscript where readers will find each of the items listed below.

Your article may not currently address all the items on the checklist. Please modify your text to include the missing information. If you are certain that an item does not apply, please write "n/a" and provide a short explanation.

Upload your completed checklist as an extra file when you submit to a journal.

In your methods section, say that you used the SPIRITreporting guidelines, and cite them as:

Chan A-W, Tetzlaff JM, Gøtzsche PC, Altman DG, Mann H, Berlin J, Dickersin K, Hróbjartsson A, Schulz KF, Parulekar WR, Krleža-Jerić K, Laupacis A, Moher D. SPIRIT 2013 Explanation and Elaboration: Guidance for protocols of clinical trials. BMJ. 2013;346:e7586

		Reporting Item	Page Number
**Administrative information**			
Title	#1	Descriptive title identifying the study design, population, interventions, and, if applicable, trial acronym	1
Trial registration	#2a	Trial identifier and registry name. If not yet registered, name of intended registry	2
Trial registration: data set	#2b	All items from the World Health Organization Trial Registration Data Set	4-16
Protocol version	#3	Date and version identifier	2
Funding	#4	Sources and types of financial, material, and other support	19
Roles and responsibilities: contributorship	#5a	Names, affiliations, and roles of protocol contributors	1,19

For peer review only

Roles and responsibilities: sponsor contact information	#5b	Name and contact information for the trial sponsor	1
Roles and responsibilities: sponsor and funder	#5c	Role of study sponsor and funders, if any, in study design; collection, management, analysis, and interpretation of data; writing of the report; and the decision to submit the report for publication, including whether they will have ultimate authority over any of these activities	19
Roles and responsibilities: committees	#5d	Composition, roles, and responsibilities of the coordinating centre, steering committee, endpoint adjudication committee, data management team, and other individuals or groups overseeing the trial, if applicable (see Item 21a for data monitoring committee)	n/a
**Introduction**			
Background and rationale	#6a	Description of research question and justification for undertaking the trial, including summary of relevant studies (published and unpublished) examining benefits and harms for each intervention	2
Background and rationale: choice of comparators	#6b	Explanation for choice of comparators	2-4
Objectives	#7	Specific objectives or hypotheses	4
Trial design	#8	Description of trial design including type of trial (eg, parallel group, crossover, factorial, single group), allocation ratio, and framework (eg, superiority, equivalence, non-inferiority, exploratory)	4
**Methods: Participants, interventions, and outcomes**			
Study setting	#9	Description of study settings (eg, community clinic, academic hospital) and list of countries where data will be collected. Reference to where list of study sites can be obtained	6
Eligibility criteria	#10	Inclusion and exclusion criteria for participants. If applicable, eligibility criteria for study centres and individuals who will	7

For peer review only

		perform the interventions (eg, surgeons, psychotherapists)	
Interventions: description	#11a	Interventions for each group with sufficient detail to allow replication, including how and when they will be administered	8
Interventions: modifications	#11b	Criteria for discontinuing or modifying allocated interventions for a given trial participant (eg, drug dose change in response to harms, participant request, or improving / worsening disease)	10
Interventions: adherance	#11c	Strategies to improve adherence to intervention protocols, and any procedures for monitoring adherence (eg, drug tablet return; laboratory tests)	10-11
Interventions: concomitant care	#11d	Relevant concomitant care and interventions that are permitted or prohibited during the trial	10-11
Outcomes	#12	Primary, secondary, and other outcomes, including the specific measurement variable (eg, systolic blood pressure), analysis metric (eg, change from baseline, final value, time to event), method of aggregation (eg, median, proportion), and time point for each outcome. Explanation of the clinical relevance of chosen efficacy and harm outcomes is strongly recommended	12-14
Participant timeline	#13	Time schedule of enrolment, interventions (including any run-ins and washouts), assessments, and visits for participants. A schematic diagram is highly recommended (see Figure)	9, Figure2
Sample size	#14	Estimated number of participants needed to achieve study objectives and how it was determined, including clinical and statistical assumptions supporting any sample size calculations	15
Recruitment	#15	Strategies for achieving adequate participant enrolment to reach target sample size	6
**Methods: Assignment of interventions (for controlled trials)**			
Allocation: sequence generation	#16a	Method of generating the allocation sequence (eg, computer-generated random numbers), and list of any factors for stratification. To reduce predictability of a random sequence, details of any planned restriction (eg, blocking) should be provided in a separate document that is unavailable to those who enrol participants or assign interventions	8

For peer review only

Allocation concealment mechanism	#16b	Mechanism of implementing the allocation sequence (eg, central telephone; sequentially numbered, opaque, sealed envelopes), describing any steps to conceal the sequence until interventions are assigned	8
Allocation: implementation	#16c	Who will generate the allocation sequence, who will enrol participants, and who will assign participants to interventions	8
Blinding (masking)	#17a	Who will be blinded after assignment to interventions (eg, trial participants, care providers, outcome assessors, data analysts), and how	8
Blinding (masking): emergency unblinding	#17b	If blinded, circumstances under which unblinding is permissible, and procedure for revealing a participant's allocated intervention during the trial	8
**Methods: Data collection, management, and analysis**			
Data collection plan	#18a	Plans for assessment and collection of outcome, baseline, and other trial data, including any related processes to promote data quality (eg, duplicate measurements, training of assessors) and a description of study instruments (eg, questionnaires, laboratory tests) along with their reliability and validity, if known. Reference to where data collection forms can be found, if not in the protocol	12-14
Data collection plan: retention	#18b	Plans to promote participant retention and complete follow-up, including list of any outcome data to be collected for participants who discontinue or deviate from intervention protocols	12-14
Data management	#19	Plans for data entry, coding, security, and storage, including any related processes to promote data quality (eg, double data entry; range checks for data values). Reference to where details of data management procedures can be found, if not in the protocol	16
Statistics: outcomes	#20a	Statistical methods for analysing primary and secondary outcomes. Reference to where other details of the statistical analysis plan can be found, if not in the protocol	16
Statistics: additional analyses	#20b	Methods for any additional analyses (eg, subgroup and adjusted analyses)	16

For peer review only

Statistics: analysis population and missing data	#20c	Definition of analysis population relating to protocol non-adherence (eg, as randomised analysis), and any statistical methods to handle missing data (eg, multiple imputation)	16
**Methods: Monitoring**			
Data monitoring: formal committee	#21a	Composition of data monitoring committee (DMC); summary of its role and reporting structure; statement of whether it is independent from the sponsor and competing interests; and reference to where further details about its charter can be found, if not in the protocol. Alternatively, an explanation of why a DMC is not needed	16
Data monitoring: interim analysis	#21b	Description of any interim analyses and stopping guidelines, including who will have access to these interim results and make the final decision to terminate the trial	16
Harms	#22	Plans for collecting, assessing, reporting, and managing solicited and spontaneously reported adverse events and other unintended effects of trial interventions or trial conduct	14
Auditing	#23	Frequency and procedures for auditing trial conduct, if any, and whether the process will be independent from investigators and the sponsor	16
**Ethics and dissemination**			
Research ethics approval	#24	Plans for seeking research ethics committee / institutional review board (REC / IRB) approval	16
Protocol amendments	#25	Plans for communicating important protocol modifications (eg, changes to eligibility criteria, outcomes, analyses) to relevant parties (eg, investigators, REC / IRBs, trial participants, trial registries, journals, regulators)	16
Consent or assent	#26a	Who will obtain informed consent or assent from potential trial participants or authorised surrogates, and how (see Item 32)	16
Consent or assent: ancillary studies	#26b	Additional consent provisions for collection and use of participant data and biological specimens in ancillary studies, if applicable	n/a
Confidentiality	#27	How personal information about potential and enrolled participants will be collected, shared, and maintained in order to protect confidentiality before, during, and after the trial	14

For peer review only

Declaration of interests	#28	Financial and other competing interests for principal investigators for the overall trial and each study site	19
Data access	#29	Statement of who will have access to the final trial dataset, and disclosure of contractual agreements that limit such access for investigators	20
Ancillary and post trial care	#30	Provisions, if any, for ancillary and post-trial care, and for compensation to those who suffer harm from trial participation	14
Dissemination policy: trial results	#31a	Plans for investigators and sponsor to communicate trial results to participants, healthcare professionals, the public, and other relevant groups (eg, via publication, reporting in results databases, or other data sharing arrangements), including any publication restrictions	20
Dissemination policy: authorship	#31b	Authorship eligibility guidelines and any intended use of professional writers	n/a
Dissemination policy: reproducible research	#31c	Plans, if any, for granting public access to the full protocol, participant-level dataset, and statistical code	20
**Appendices**			
Informed consent materials	#32	Model consent form and other related documentation given to participants and authorised surrogates	n/a
Biological specimens	#33	Plans for collection, laboratory evaluation, and storage of biological specimens for genetic or molecular analysis in the current trial and for future use in ancillary studies, if applicable	n/a

The SPIRIT checklist is distributed under the terms of the Creative Commons Attribution License CC-BY-ND 3.0. This checklist was completed on 07. October 2020 using https://www.goodreports.org/, a tool made by the EQUATOR Network in collaboration with Penelope.ai

For peer review only
